# Knock knock, who's there? Identifying wild species‐specific fish sounds with passive acoustic localization and random forest models

**DOI:** 10.1111/jfb.70294

**Published:** 2025-12-03

**Authors:** Darienne Lancaster, Xavier Mouy, Dana Haggarty, Francis Juanes

**Affiliations:** ^1^ University of Victoria Department of Biology Victoria British Columbia Canada; ^2^ Woods Hole Oceanographic Institution Applied Ocean Physics and Engineering Department Woods Hole Massachusetts USA; ^3^ Fisheries and Oceans Canada Pacific Biological Station Nanaimo British Columbia Canada

**Keywords:** animal communication, fish sounds, localization, passive acoustic monitoring (PAM), rockfish, Sebastes

## Abstract

Passive acoustic monitoring (PAM) is a useful non‐destructive tool for evaluating species presence, diversity and abundance. However, in marine environments, a dearth of tools and methods for identifying wild, species‐specific fish calls makes quantitative PAM assessments for specific fish species challenging. We tested a novel passive acoustic localization array with paired audio/video for identifying wild, species‐specific fish sounds in a high‐diversity region of British Columbia, Canada. We then used random forest models incorporating 47 sound features to test the feasibility of differentiating species‐specific fish calls. We identified calls for eight soniferous fish species, five of which had never been documented or described, including vermillion (*Sebastes miniatus*), canary (*S. pinniger*), and black rockfish (*S. melanops*). Random forest models were able to differentiate fish knocks and grunts to the species level with high accuracy (80% for knocks, 88% for grunts). The models struggled to differentiate species knocks when sample sizes were low. The Gini impurity index and partial dependence probability plots showed species‐specific differences in call features that measure low frequencies and central frequencies. We also provide a comprehensive set of species‐specific call characteristics for 47 sound features which can be used to parameterize fish sound detectors. Our study outlines a robust method for collecting and differentiating wild species‐specific fish sounds from a high‐diversity region with many closely related soniferous fish species. This research can be used to design a species‐specific fish sound detector for quantitative estimates of species presence, diversity and range. These adaptable methods can also be applied elsewhere using the same 47 sound features and random forest models to identify species‐specific fish sound parameters.

## 1 INTRODUCTION

Passive acoustic monitoring (PAM) is a non‐destructive tool that is growing in popularity in marine and fisheries research. Over 1000 species of fishes are known to be soniferous (Looby et al., 2021). However, the challenge of identifying species‐specific calls in the wild limits our ability to conduct assessments of species diversity and presence.

Many fish species calls have been documented in laboratory studies but few studies have identified species‐specific sounds in the wild (Amorim et al., 2013; Riera et al., 2020, 2018; Širović & Demer, 2009; Song et al., 2024; Zhang et al., 2015). Laboratory studies can determine if fish are soniferous, but recordings in aquaria are not always representative of wild sounds because aquarium acoustics alter sound features (Jézéquel et al., 2022). Laboratory studies can also struggle to elicit calls from soniferous fish (Nichols, 2005).

Many PAM studies are conducted in marine field environments, but they are usually limited in their ability to identify calls to specific fish and focus on soundscape level analyses by applying acoustic indices like the Acoustic Diversity Index (ADI) (Davies et al., 2020; McWilliam & Hawkins, 2013; Minello et al., 2021; Pieretti & Danovaro, 2020; Rice et al., 2017). Most of these acoustic indices were developed in terrestrial environments (Pekin et al., 2012; Pieretti et al., 2011; Villanueva‐Rivera et al., 2011), and cannot differentiate species calls in marine environments which occupy narrow, overlapping frequency bands (Dimoff et al., 2021; Minello et al., 2021). Some studies identify fish sound diversity, but they rely on complementary sampling methods like fishing to infer relationships between calls and species (Desiderà et al., 2019; Hin‐Kiu et al., 2011; Puebla‐Aparicio et al., 2023; Širović et al., 2009; Souza Jr et al., 2023; Wang et al., 2017). Few studies precisely tie fish sounds to specific species in the wild or describe the sound characteristics needed to create species‐specific fish sound detectors (Mouy et al., 2023, 2018).

Knowledge of terrestrial species calls is extensive and PAM is often used to monitor species presence and diversity in terrestrial domains (Acevedo & Villanueva‐Rivera, 2006; Hoefer et al., 2023; Skalak et al., 2012; Wimmer et al., 2013). Marine mammal vocalizations are also well documented (Antunes et al., 2011; Frasier et al., 2021; Schall & Parcerisas, 2022). However, we lack the same level of understanding for most fish species (Mouy et al., 2023, 2018). PAM has many advantages compared to classic fisheries monitoring like SCUBA, hook and line, or video surveys. PAM is particularly useful for long‐term monitoring as most acoustic recorders require minimal battery power and can record for months. PAM recorders are low cost (e.g. ~US$150, HydroMoth from Open Acoustic Devices [Lamont et al., 2022] or ~UD$3000, SoundTrap from Ocean Instruments NZ, New Zealand), effective in low visibility (e.g. deep water, nighttime) and can continuously monitor in remote regions or overwinter. PAM is a promising monitoring method, but more tools and techniques are required to decipher fish species level acoustic information.

Our research advances the applications of PAM in marine environments by identifying wild species‐specific fish sounds and developing a method to define unique species sound characteristics for use in fish sound detectors. We hypothesized that fish calls identified to the species level could be differentiated from one another through an analysis of 47 sound features (e.g. peak frequency, sound duration, etc.). Mouy et al. (2023) found some measurable differences in sound characteristics for a small sample of copper rockfish (*Sebastes caurinus*), quillback rockfish (*Sebastes maliger*) and lingcod (*Ophiodon elongatus*). We build on this research through a study of rocky reef fish calls in British Columbia, Canada. There are 41 species of rockfish (*Sebastes* spp.) as well as lingcod, kelp greenling (*Hexagrammos decagrammus*) and many other fish species that occupy rocky reef habitats in British Columbia. Many of these closely related species overlap habitats (Burford and Bernardi 2008; Love et al., 2002; Schwenke et al., 2018; Tolimieri et al., 2009) and are often present in high numbers throughout coastal areas, which adds to the challenge of differentiating their calls. In our study we manually match localized sounds to fish in underwater video and use random forest models to test if fish knocks and grunts can be differentiated to the species level using sound features. We also describe distinctive sound features for the fish species we identified that can be used to parameterize species‐specific fish sound detectors. Our study provides a novel method for identifying and describing wild species‐specific fish sounds, which advances PAM from a basic soundscape tool towards a standalone method of quantitatively monitoring fish diversity and presence.

## 2 MATERIALS AND METHODS

### 2.1 Data collection and processing

**FIGURE 1:**
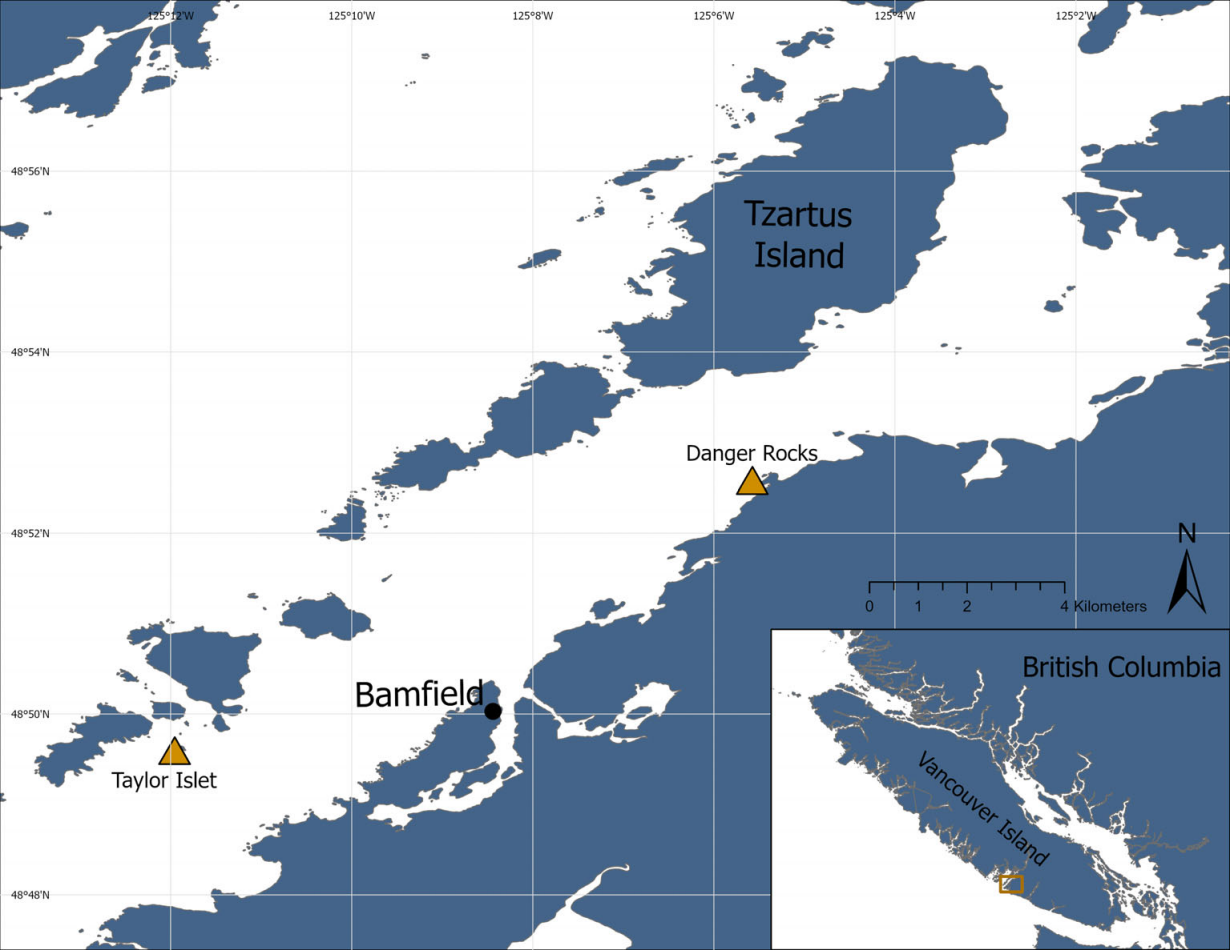
Site map of passive acoustic localization array deployment locations (yellow triangles).

Acoustic data were collected using an audio/video localization array near Bamfield Marine Science Centre in Barkley Sound, BC, Canada from August to September 2022 (Mouy et al., 2018; Figure 1). The localization array was deployed for 12 days at Taylor Islet and 8 days at Danger Rocks.

**FIGURE 2:**
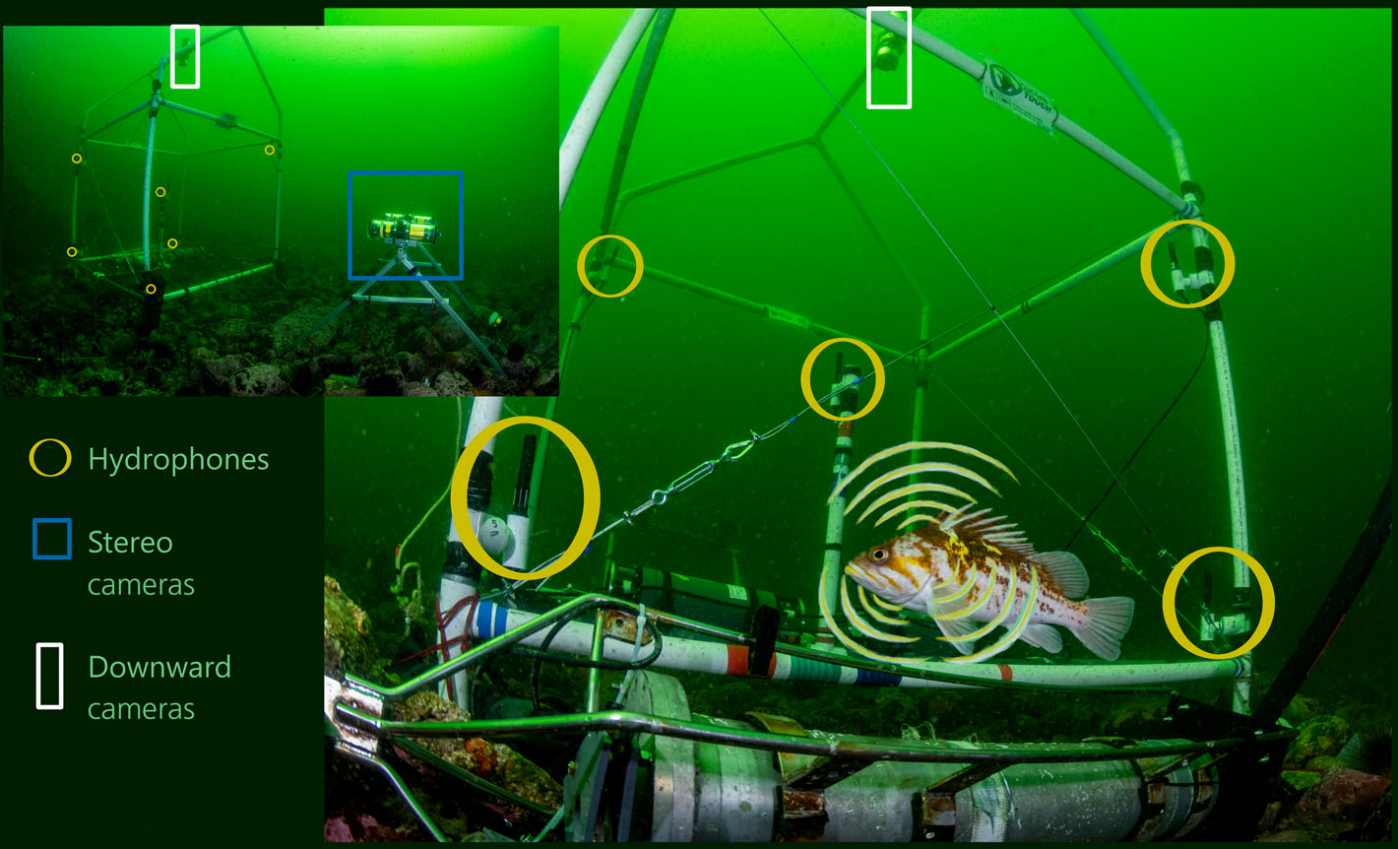
Localization array with visualization of copper rockfish (*Sebastes caurinus*) calling near hydrophones. (images, Shane Gross; graphics, Darienne Lancaster).

The array was deployed by divers at sites with high fish diversity and abundance. The array used an AMAR‐G3R4.3 acoustic recorder (JASCO Applied Sciences) with 6M36‐V35‐100 omnidirectional hydrophones (GeoSpectrum Technologies Inc.) for recording and localizing fish calls, and a horizontal stereo‐camera and downward facing camera for identifying fish species (Figure 2).

Hydrophones were arranged within the array to maximize three‐dimensional (3D) localization accuracy. For full details on large array specifications and AMAR recording parameters see Mouy et al. (2023). Video was recorded with underwater FishCams designed for maximum battery life, see Mouy et al. (2020) for full specifications. Cameras recorded 5‐min h264 video files at 10 frames per second and 1600 × 1200 resolution continuously from 6 AM to 9 PM Pacific Standard Time (~sunrise/sunset). Cameras emitted a beep sequence every 4 h to allow for time synchronization with acoustic recordings.

Acoustic data were processed in Python (ver. 3.9) in a three‐step process: (1) identification of acoustic transients on the arrays central hydrophone (Figure 2), (2) time‐difference of arrival estimation using cross‐correlation and (3) 3D localization of sound source (Mouy et al., 2023). Localized sounds were filtered in R (ver. 3.4.4) to retain sounds occurring within the downward facing camera field of view (FOV; 2 m width × 2 m length × 3 m height) with localization uncertainties of <2 m. Sounds were manually annotated in RavenPro 1.6 and identified as fish sound, unknown sound or noise. Fish sounds were further classified based on frequency and duration characteristics into knocks (short [~1–2 ms], low‐frequency [<1000 kHz] pulses), grunts (longer, low‐frequency sounds with modulation and/or harmonic structures), and other (fish sounds not defined as knock or grunt). Clock drift between audio/video data was quantified by manually analysing buzzer timestamps in RavenPro every 4 h across the full deployment. Clock drift times were applied to video file time stamps to correctly match video files to audio files. Coordinate plots with localization uncertainty bars were created for each sound and used to visually identify the sound source (e.g. calling fish) from the paired video footage. Video data were annotated in EventMeasure software (SeaGIS Pty Ltd). Calling fish visible in the downward facing camera FOV were identified to the lowest possible taxonomic level. All identified calling fish were given a species identification (ID) confidence rating from 1 to 3 to account for any difficulties visually identifying species or when localization coordinates were not perfectly matched to fish location. A confidence score of 1 represented >95% identification certainty (distinctive species marking and movement patterns were clearly visible and calling fish position within the camera FOV matched localization coordinates), confidence score of 2 represented 75% to 95% identification certainty (one of either distinctive species markings or movement patterns were visible and/or calling fish position within the camera FOV was within <0.5 m radius of localization coordinates) and confidence score of 3 represented <75% identification certainty (distinctive species markings or movement patterns were suggestive of a particular species but could not be fully verified and/or calling fish position within the camera FOV was beyond a 0.5 m radius of localization coordinates). The forward‐facing stereo cameras were used to identify species when calling fish were not visible in the downward facing camera. Localized sounds with no fish visible within 0.75 m of sound source coordinates were excluded from the analysis. RavenPro selection tables of fish sounds were processed in Python using the ecosound package to create a set of 45 sound features using a time/frequency box containing 95% of sound energy within each selection box (Table 1). For full description see Mouy et al. (2024). These 45 sound features were selected for their ability to differentiate species‐specific marine animal sounds (Cortopassi, 2006; Mellinger & Bradbury, 2007). We also included high‐frequency and low‐frequency measurements from RavenPro selection boxes for a total of 47 sound features. All fish calls were truncated to 25 Hz to avoid a low‐frequency noise band below that threshold. Spectrograms of representative grunts and knocks for each species were created in Python using the function specgram from package Matplotlib.pyplot. We calculated the mean and standard deviation of high frequency, low frequency, peak frequency, frequency bandwidth and time duration for all grunts and knocks with a species ID confidence of 1 for each species (included ID confidence 2 for Lingcod and Kelp Greenling as no level 1 confidence recorded).

**TABLE 1:** Description of 47 sound features used for species‐specific sound classification.

	Feature	Units	Description	Calculated from
F1	Peak frequency	Hz	Frequency of highest amplitude peak	Spectral envelope
F2	Frequency bandwidth	Hz	Maximum frequency—minimum frequency	Spectral envelope
F3	Frequency bandwidth 90%	Hz	F8–F5	Spectral envelope
F4	Frequency 5%	Hz	Frequency at which cumulative energy reaches 5% of total energy (measured from lowest frequencies upward).	Spectral envelope
F5	Frequency 25%	Hz	Frequency at which cumulative energy reaches 25% of total energy (measured from median downward). Also known as quartile 1.	Spectral envelope
F6	Frequency 50%	Hz	Frequency at which cumulative energy reaches 50% of total energy (measured from lowest frequencies upward). Also known as median.	Spectral envelope
F7	Frequency 75%	Hz	Frequency at which cumulative energy reaches 75% of total energy (measured from median upward). Also known as quartile 3.	Spectral envelope
F8	Frequency 95%	Hz	Frequency at which cumulative energy reaches 95% of total energy (measured from lowest frequencies upward).	Spectral envelope
F9	Frequency bandwidth 50%	Hz	F7–F5	Spectral envelope
F10	Spectral asymmetry	None	(F5 + F7 − 2F6)/(F5 + F7)	Spectral envelope
F11	Spectral concentration	Hz	Difference of maximum and minimum frequencies in cumulative sum of ranked amplitude values	Spectral envelope
F12	Frequency standard deviation	Hz	Standard deviation of spectral envelope (about the mean)	Spectral envelope
F13	Frequency kurtosis	None	Kurtosis of spectral envelope	Spectral envelope
F14	Frequency skewness	None	Skewness of spectral envelope	Spectral envelope
F15	Spectral entropy	Bit	Shannon entropy of the spectral envelope	Spectral envelope
F16	Spectral flatness	None	Tends to 1 for noisy signal and to 0 for pure tone signal	Spectral envelope
F17	Spectral roughness	None	Total curvature of the spectral envelope	Spectral envelope
F18	Frequency centroid	Hz	Frequency of centre of mass in spectral envelope (weighted average of all frequencies within a spectrum)	Spectral envelope
F19	Overall frequency peak	Hz	Frequency of maximum amplitude value in spectrogram	Spectrogram
F20	Mean median frequency	Hz	Mean of median frequencies calculated for each time slice of spectrogram	Spectrogram
F21	Median frequency standard deviation	Hz	Standard deviation of median frequencies calculated for each time slice of spectrogram	Spectrogram
F22	Mean spectral entropy	Bit	Mean of Shannon entropy calculated for each time slice of spectrogram	Spectrogram
F23	Spectral entropy standard deviation	Bit	Standard deviation of Shannon entropy calculated for each time slice of spectrogram	Spectrogram
F24	Mean frequency shift	Hz	Mean of differences between median frequencies of consecutive spectrogram time slices	Spectrogram
F25	Fraction of upsweep frequency	%	Percent of time median frequency increases from one spectrogram time slice to the next	Spectrogram
F26	Signal to noise ratio	dB	Calculated from ratio of maximum and 25th percentile energy values in spectrogram	Spectrogram
F27	Time of energy peak	s	Time of highest amplitude peak	Temporal envelope
F28	Relative time of energy peak	%	Ratio of F27 and F29	Temporal envelope
F29	Duration	s	Length of temporal envelope	Temporal envelope
F30	Time 5%	s	Time at which cumulative energy reaches 5% of total energy	Temporal envelope
F31	Time 25%	s	Time at which cumulative energy reaches 25% of total energy	Temporal envelope
F32	Time 50%	s	Time at which cumulative energy reaches 50% of total energy	Temporal envelope
F33	Time 75%	s	Time at which cumulative energy reaches 75% of total energy	Temporal envelope
F34	Time 95%	s	Time at which cumulative energy reaches 95% of total energy	Temporal envelope
F35	Duration 50%	s	F33–F31	Temporal envelope
F36	Duration 90%	s	F34–F30	Temporal envelope
F37	Temporal asymmetry	None	(F31 + F33 − 2F32)/(F31 + F33)	Temporal envelope
F38	Temporal concentration	s	Difference of maximum and minimum times in cumulative sum of ranked amplitude values	Temporal envelope
F39	Time standard deviation	s	Standard deviation of temporal envelope	Temporal envelope
F40	Time kurtosis	None	Kurtosis of temporal envelope	Temporal envelope
F41	Time skewness	None	Skewness of temporal envelope	Temporal envelope
F42	Temporal entropy	Bits	Shannon entropy of a temporal envelope	Temporal envelope
F43	Temporal flatness	None	Flatness of temporal envelope. Tends towards 1 for noisy signal and towards 0 for pure tone signal	Temporal envelope
F44	Temporal roughness	None	Roughness of temporal envelope	Temporal envelope
F45	Temporal centroid	s	Time of centre of mass in temporal envelope	Temporal envelope
F46	High frequency^a^	Hz	Highest frequency based on selection box boundary	Selection box
F47	Low frequency^a^	Hz	Lowest frequency based on selection box boundary	Selection box

^a^ Denotes features not measured from 95% energy envelopes. Table adapted from Mouy et al. (2024).

### 2.2 Random forest classification

Sound features were used to classify fish sounds to the species level using a random forest ensemble classification technique in R (see Tables S3–S5 for full sound feature results). Random forest uses many independently grown decision trees to partition data into classes (i.e., species) based on the values of a single feature at each split (node). The models are considered random as each tree is built on a subsample of the training dataset and the features used to split data are chosen at random for each node. We used the package randomForest to partition fish grunts and knocks into species classes (Breiman, 2001). We used 2000 decision trees with six random features per split per tree to maximize the stability of the conditional mean while minimizing computation time. Fish sound feature data were split into training (70%) and testing (30%) datasets. Fish sounds with a confidence rating of 1 were used for most species to maximize ID confidence. Fish sounds with a confidence of 1 and 2 were included for lingcod knocks and quillback rockfish grunts to boost the small sample sizes for those classes. Vermillion rockfish (*Sebastes miniatus*) and kelp greenling knocks were not included in the random forest knock model because of low sample size (<5) and low species ID confidence scores, respectively. Canary rockfish (*Sebastes pinniger*) grunts were not included in the grunt model because of low sample size (<5). Fish grunt and knock sounds were included in separate models to avoid confounding the model with intra‐species differences attributed to grunt vs. knock sound features. A model with both grunts and knocks was tested but model performance was poor. To investigate the impact of unbalanced classes on the random forest models we tested models with upsampling to balance classes and with class weights using the ranger package (Wright & Ziegler, 2017). The upsampled model predictions showed extremely high accuracy, precision, recall and F1 scores for all classes, suggesting artificially inflated performance. The class‐weighted models did not perform better than the unweighted/unbalanced models so we only present results from the original dataset without class weighting or upsampling. We calculated variable importance plots showing mean decrease in Gini impurity index and mean decrease in accuracy for each model. The Gini impurity index is a measure of how well a feature splits classes at each node and mean decrease in accuracy expresses how much accuracy the model loses when each variable is excluded (Breiman, 2001). Model performance on the test dataset was evaluated with precision, recall and F1 scores for each class and accuracy scores for overall model performance. Precision is a measure of false positives given in Equation (1), where tp *=* true positives and fp *=* false positives.

(1) $$\text{Precision}=\frac{\mathrm{tp}}{\mathrm{tp}+\mathrm{fp}}$$

Recall is a measure of false negatives given in Equation (2), where tp *=* true positives and fn *=* false negatives.

(2) $$\begin{array}{c} \text{Recall}=\frac{\mathrm{tp}}{\mathrm{tp}+\mathrm{fn}} \end{array}$$

F1 score is a metric (Equation 3) that combines both precision and recall to evaluate overall performance.

(3) $$\begin{array}{c} F1=2\times \frac{\text{precision}\times \text{recall}}{\text{precision}+\text{recall}\;} \end{array}$$

Partial dependence probability (PDP) plots were created for each model using the partial function from package pdp (Greenwell, 2017). PDP plots illustrate how the six top sound features (based on Gini impurity index scores) were used to differentiate species sounds. Uniform manifold approximation and projection (UMAP) plots were created using the package umap to visualize different species clusters in two‐dimensional space and validate random forest model performance (Healy & McInnes, 2024). UMAP is an unsupervised dimension reduction technique similar to principal component analysis used for visualizing high‐complexity data by preserving the local and global structure of high‐dimensional data (Healy & McInnes, 2024).

## 3 RESULTS

**FIGURE 3:**
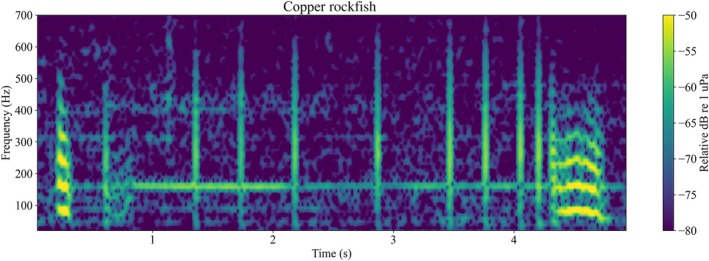
Example of grunt‐knock vocalization sequence for a copper rockfish (*Sebastes caurinus*).

We identified 1107 fish calls for eight species. Over half of the fish calls were identified with high species ID confidence (*n* = 613). We identified 452 calls with moderate ID confidence and 42 calls with low ID confidence. All fish sounds were in a low‐frequency band (<1000 kHz). Knocks were 4.6 times more abundant than grunts. Knocks and grunts often occurred in repetitive sequences (Figure 3) rather than in isolation. Other fish sounds (i.e. non‐grunt/knock) occurred infrequently (*n* = 10) and were only documented for three species (Table S6).

### 3.1 Knock results

**FIGURE 4:**
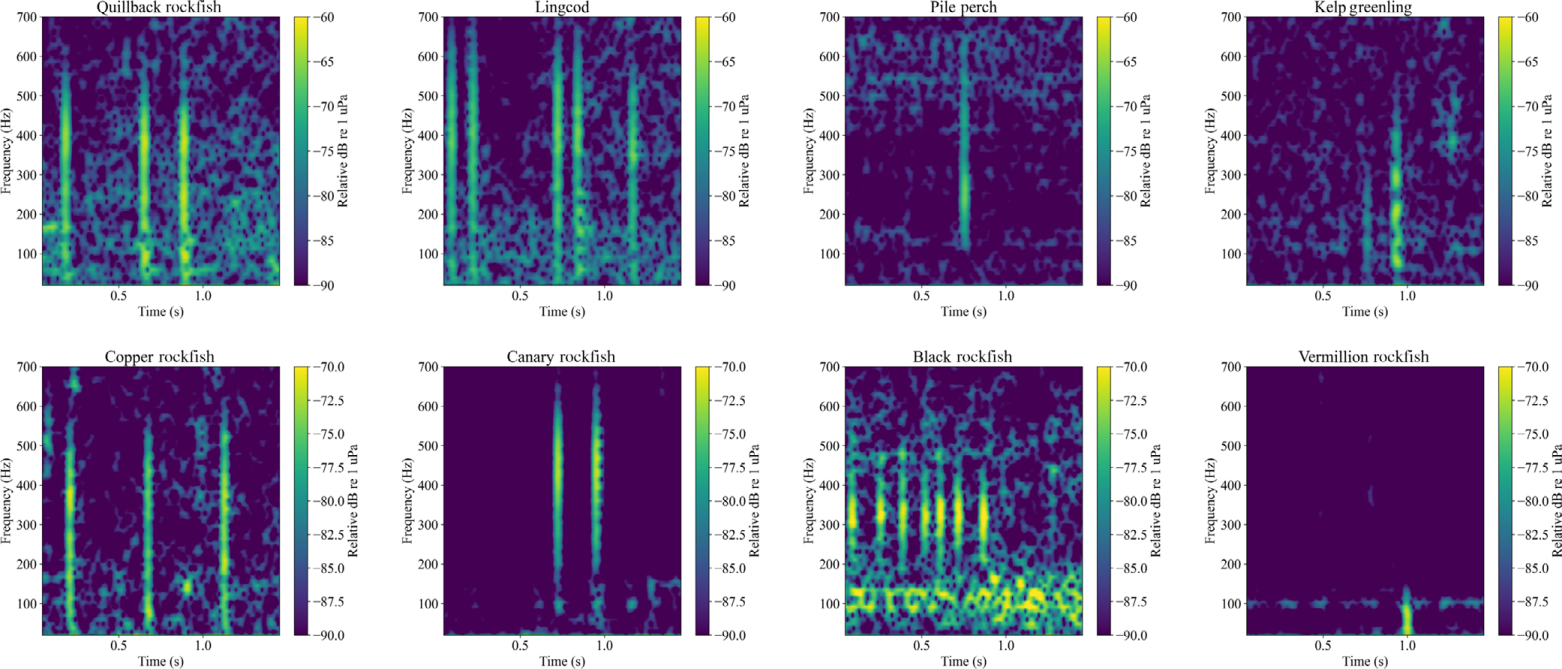
Example knocks for all identified knocking species. All wav files amplified by factor 10. All spectrograms calculated with NFFT = 2048 and noverlap = 2000. Full species scientific names: black rockfish (*Sebastes melanops*), canary rockfish (*Sebastes pinniger*), copper rockfish (*Sebastes caurinus*), lingcod (*Ophiodon elongatus*), pile perch (*Rhacochilus vacca*), quillback rockfish (*Sebastes maliger*), kelp greenling (*Hexagrammos decagrammus*), vermillion rockfish (*Sebastes miniatus*).

Canary and black rockfish knocks were the highest frequency sounds, with the peak frequency occurring at ~350 Hz. Vermillion rockfish knocks were the lowest frequency sounds, with the peak frequency occurring at ~86 Hz (Table 2 and Figure 4). Black and vermillion rockfish have distinctive knocks that cover a smaller range of frequences than other species.

**TABLE 2:** Summary of knock features (mean and standard deviation) for each species.

Species	*n*	Knock high frequency (Hz)	Knock low frequency (Hz)	Knock peak frequency (Hz)	Knock bandwidth (Hz)	Knock duration (s)
Black rockfish	28	526.79 ± 136.26	183.36 ± 66.01	341.54 ± 129.26	345.30 ± 104.17	0.12 ± 0.05
Canary rockfish	253	756.35 ± 126.31	104.59 ± 62.06	356.17 ± 165.39	649.30 ± 132.99	0.14 ± 0.04
Copper rockfish	147	590.48 ± 133.43	40.29 ± 28.70	189.96 ± 120.24	550.29 ± 142.13	0.14 ± 0.04
Lingcod	52	694.64 ± 111.28	68.15 ± 66.88	308.15 ± 111.54	627.10 ± 147.18	0.15 ± 0.03
Pile Perch	15	653.80 ± 154.20	47.63 ± 33.85	269.28 ± 162.48	606.38 ± 146.74	0.16 ± 0.02
Quillback rockfish	44	569.87 ± 101.85	66.25 ± 51.21	232.95 ± 124.57	504.23 ± 134.05	0.13 ± 0.03
Vermillion rockfish	2	159.80 ± 22.60	36.98 ± 5.65	85.92 ± 22.00	121.25 ± 27.65	0.16 ± 0.01

*Note*: Only sounds with an ID confidence of 1 (high) were included for most species. Sounds with ID confidence 2 (moderate) were included for lingcod as no high confidence knocks were recorded. Kelp greenling (*Hexagrammos decagrammus*) knocks were not included as ID confidence for all knocks was low. Full species scientific names: black rockfish (*Sebastes melanops*), canary rockfish (*Sebastes pinniger*), copper rockfish (*Sebastes caurinus*), lingcod (*Ophiodon elongatus*), pile perch (*Rhacochilus vacca*), quillback rockfish (*Sebastes maliger*), vermillion rockfish (*Sebastes miniatus*).

#### 3.1.1 Random forest knock model results

The random forest model performed well for classifying knocks for specific fish species. The model had an out of bag (OOB) error rate of 18% for the training data and overall accuracy of 80%. The model identified black and canary rockfish knocks with high precision and recall, and F1 scores of 0.93 (Table 3). Recall for copper rockfish knocks was high (0.93) but precision was lower (0.68) with multiple false positives. The model showed moderately high precision for lingcod knock classification but misclassified multiple calls (recall = 0.47, *F* score = 0.58). Quillback rockfish call classification showed low precision and recall, and had an overall F1 score of 0.22. Pile perch calls were never predicted in the model test.

**TABLE 3:** Random forest results from knock test dataset.

Knock class	Precision	Recall	F1 score	Prevalence	Detection rate	Detection prevalence	Balanced accuracy	*n*
Black rockfish	1.00	0.88	0.93	0.05	0.04	0.04	0.94	8
Canary rockfish	0.91	0.95	0.93	0.47	0.45	0.49	0.93	75
Copper rockfish	0.68	0.93	0.78	0.27	0.25	0.37	0.88	43
Lingcod	0.78	0.47	0.58	0.09	0.04	0.06	0.73	15
Pile perch	N/A	0.00	N/A	0.03	0.00	0.00	0.50	4
Quillback rockfish	0.40	0.15	0.22	0.08	0.01	0.03	0.57	13

*Note*: Pile perch were never predicted in the model test so not applicable (N/A) values are present for precision and F1 score. Full species scientific names: black rockfish (*Sebastes melanops*), canary rockfish (*Sebastes pinniger*), copper rockfish (*Sebastes caurinus*), lingcod (*Ophiodon elongatus*), pile perch (*Rhacochilus vacca*), quillback rockfish (*Sebastes maliger*).

**FIGURE 5:**
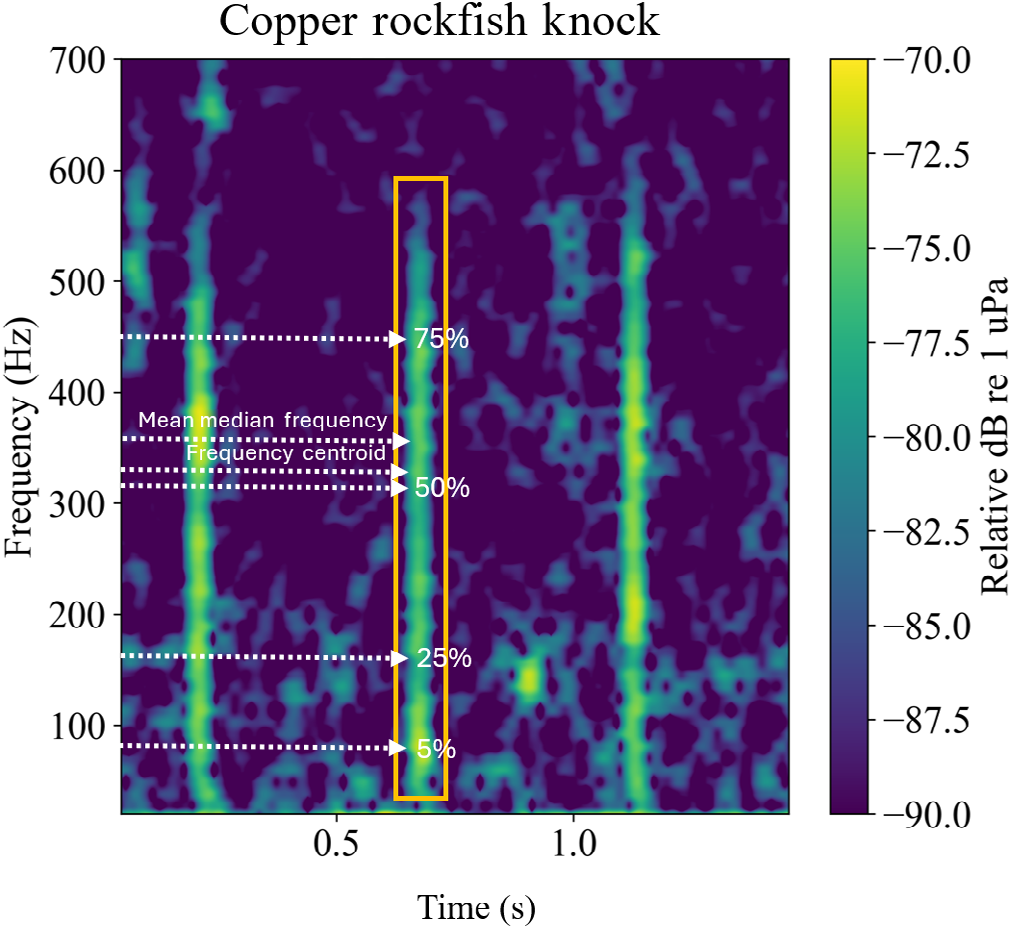
Visualization of the location of the top six sound features for differentiating fish knocks. The example spectrogram shows locations of frequency features on a single copper rockfish (*Sebastes caurinus*) knock. Percentages represent frequency features (i.e. frequency 25%). Sound selection boxes are shown in yellow.

The top three sound features for differentiating fish knocks using the Gini impurity index were all measures of frequency central tendencies (frequency 50%, frequency centroid and mean median frequency; Figure 5 and Table S3). See Table 1 for full descriptions of sound features. The top differentiating variable was frequency 50%, also called median frequency, which indicates the midpoint of total energy within a fish call and assesses a call's central tendency with limited outlier influence (Figure 5).

**FIGURE 6:**
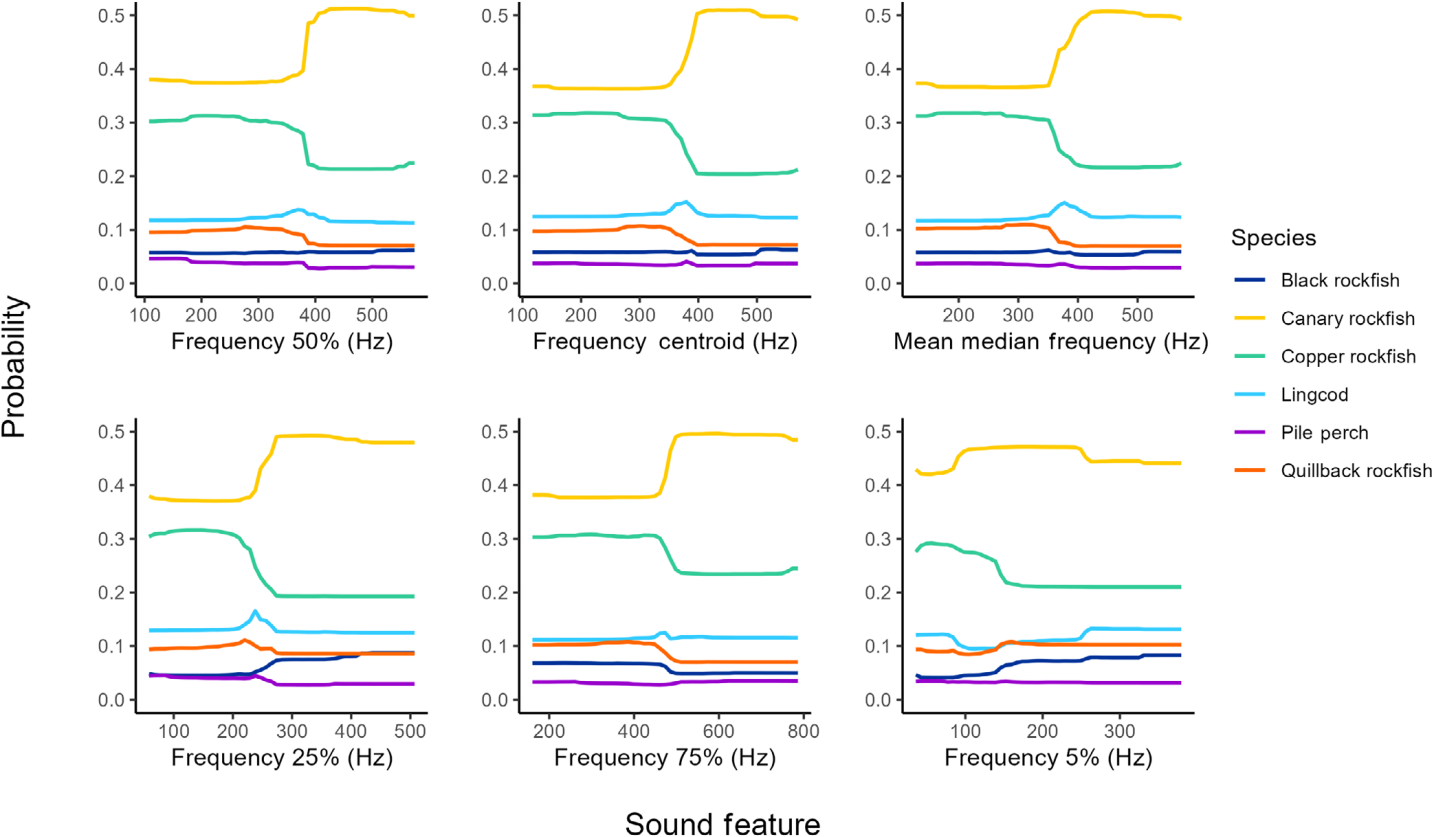
Partial dependence probability plots for the top six knock sound features (Gini impurity index). Full species scientific names: black rockfish (*Sebastes melanops*), canary rockfish (*Sebastes pinniger*), copper rockfish (*Sebastes caurinus*), lingcod (*Ophiodon elongatus*), pile perch (*Rhacochilus vacca*), quillback rockfish (*Sebastes maliger*).

PDP plots showed the probability of a call being made by a canary rockfish increased when frequency 50%, frequency centroid and mean median frequency were higher than ~400 Hz (Figure 6). The opposite trend was observed in copper rockfish, with call probability decreasing when frequency 50%, frequency centroid and mean median frequency were higher than ~400 Hz (Figure 6).

**FIGURE 7:**
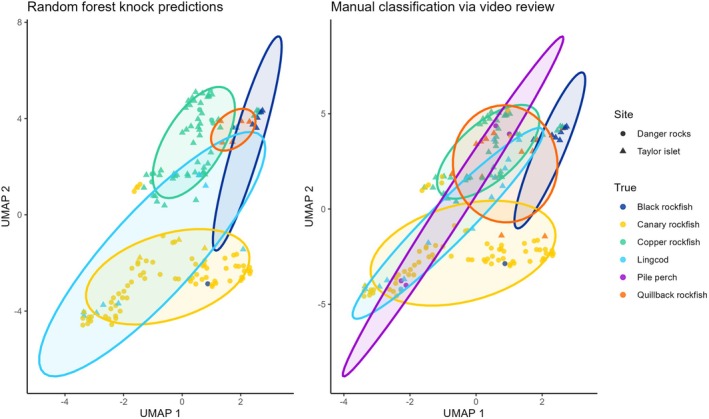
Uniform manifold approximation and projection visualization of knock sound feature groupings coloured by species class with 70% confidence level ellipses. Full species scientific names: black rockfish (*Sebastes melanops*), canary rockfish (*Sebastes pinniger*), copper rockfish (*Sebastes caurinus*), lingcod (*Ophiodon elongatus*), pile perch (*Rhacochilus vacca*), quillback rockfish (*Sebastes maliger*).

The probability of a call being a black rockfish increased when frequency 25% and 75% values were between ~250 and ~500 Hz. Frequency 25% and 75%, also called quartile 1 and 3, indicate the frequency below which 25% and 75% of total sound energy occurs. These features are useful for detecting differences in sound shape and spread, and can indicate energy skewedness across a sound's frequency range. Quillback rockfish generally showed similar trends to copper rockfish except for frequency 5%, where quillback rockfish call probability increased above ~150 Hz and copper rockfish call probability decreased. Frequency 5% is a measure of low frequency that is less influenced by outliers and manual selection box errors than a basic low frequency measure. Lingcod call probability showed a pattern of probability spikes at various sound feature values, suggesting there may be a narrow range of values that could help differentiate lingcod calls from other species calls with similar sound features. Pile perch sample size was small and the model struggled to classify their knocks. UMAP dimension reduction plots did not show any site‐based trends (i.e. model is not classifying species calls based on site background noise; Figure 7). Canary, black and copper rockfish clusters show clear separation from each other. Lingcod, quillback and pile perch knocks overlap with multiple species.

### 3.2 Grunt results

**FIGURE 8:**
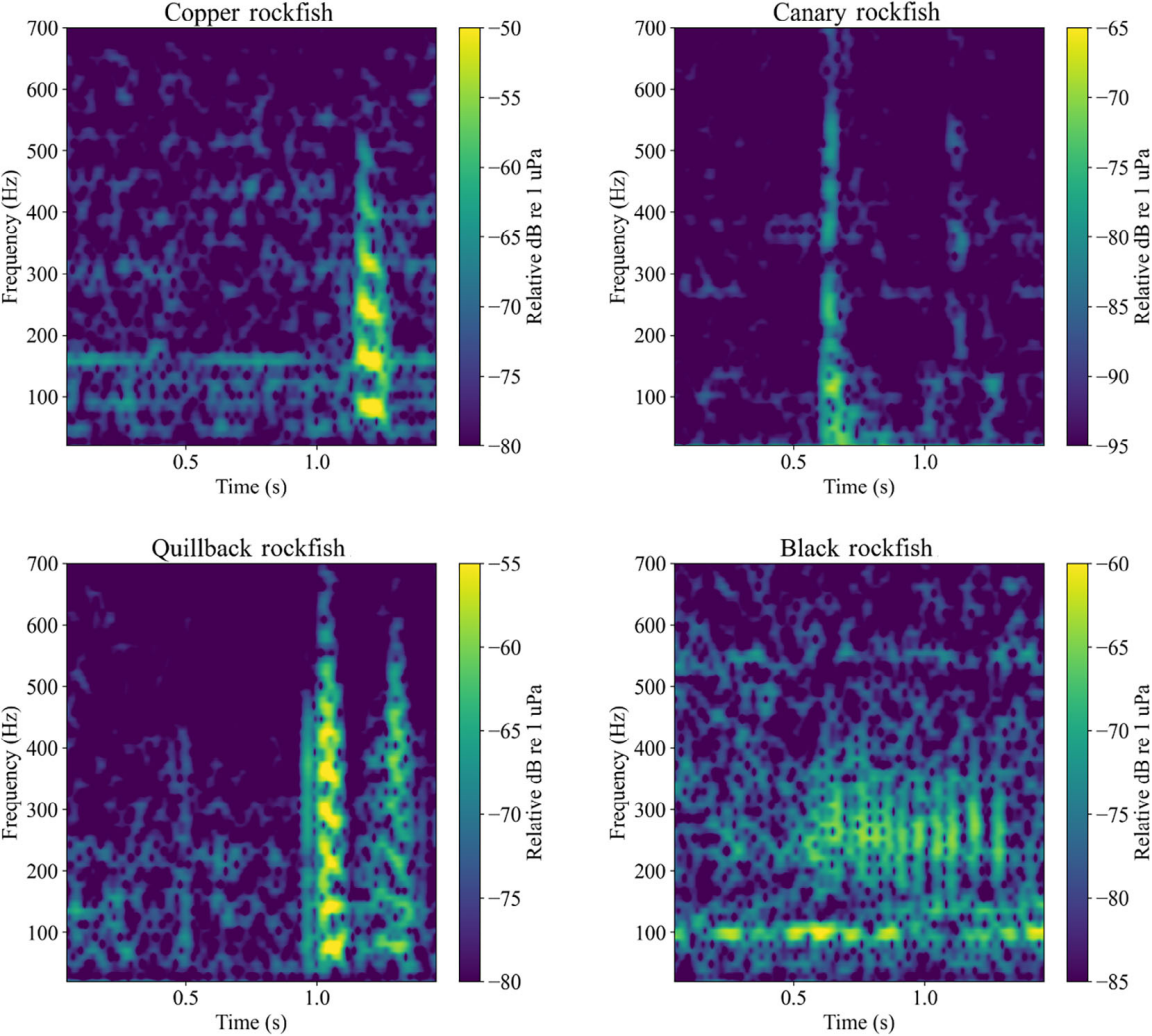
Example grunts for all identified grunting species. All wav files amplified by factor 10. All spectrograms calculated with NFFT = 2048 and noverlap = 2000. The canary rockfish grunt sound occurred during a feeding lunge so may represent a passive incidental sound rather than an active, communication sound. Full species scientific names: black rockfish (*Sebastes melanops*), canary rockfish (*Sebastes pinniger*), copper rockfish (*Sebastes caurinus*), quillback rockfish (*Sebastes maliger*).

Black rockfish grunts showed the highest mean peak frequency (~360 Hz) and copper rockfish grunts showed the lowest mean peak frequency (~110 Hz) (Table 4, Figure 8). Copper and quillback rockfish grunts showed similar frequency and duration characteristics, and both had visible harmonics (i.e. repeating bands of sound above the fundamental frequency). Black rockfish grunts were distinctive from other recorded grunts with a smaller range of frequencies (i.e. bandwidth), no visible harmonics and a longer duration (>1 s). Copper and quillback rockfish grunts sounded like a croak and black rockfish grunts sounded like a growl (see data repository for sample audio files). Two canary rockfish grunts were recorded but they occurred during a feeding movement so this sound may represent a passive rather than active sound (Table 4 and Figure 8).

**TABLE 4:** Summary of grunt sound features (mean and standard deviation) for each species.

Species	*n*	Grunt high frequency (Hz)	Grunt low frequency (Hz)	Grunt peak frequency (Hz)	Grunt bandwidth (Hz)	Grunt duration (s)
Black rockfish	45	525.46 ± 95.45	182.64 ± 55.38	361.81 ± 102.27	342.33 ± 77.09	1.23 ± 0.86
Canary rockfish	2	643.19 ± 35.78	35.65 ± 0.00	39.06 ± 0.00	609.55 ± 33.16	0.32 ± 0.02
Copper rockfish	50	540.51 ± 131.67	33.21 ± 16.32	107.51 ± 54.65	507.33 ± 135.72	0.36 ± 0.37
Quillback rockfish	9	627.12 ± 117.29	30.07 ± 7.73	188.47 ± 110.29	597.80 ± 114.23	0.21 ± 0.08

*Note*: Only sounds with an ID confidence of 1 (high) were included. Full species scientific names: black rockfish (*Sebastes melanops*), canary rockfish (*Sebastes pinniger*), copper rockfish (*Sebastes caurinus*), quillback rockfish (*Sebastes maliger*).

#### 3.2.1 Random forest grunt model results

The grunt random forest model performed well and classified grunts to specific species with high accuracy (88% on test dataset) and a 6% OOB error rate on the training dataset.

**FIGURE 9:**
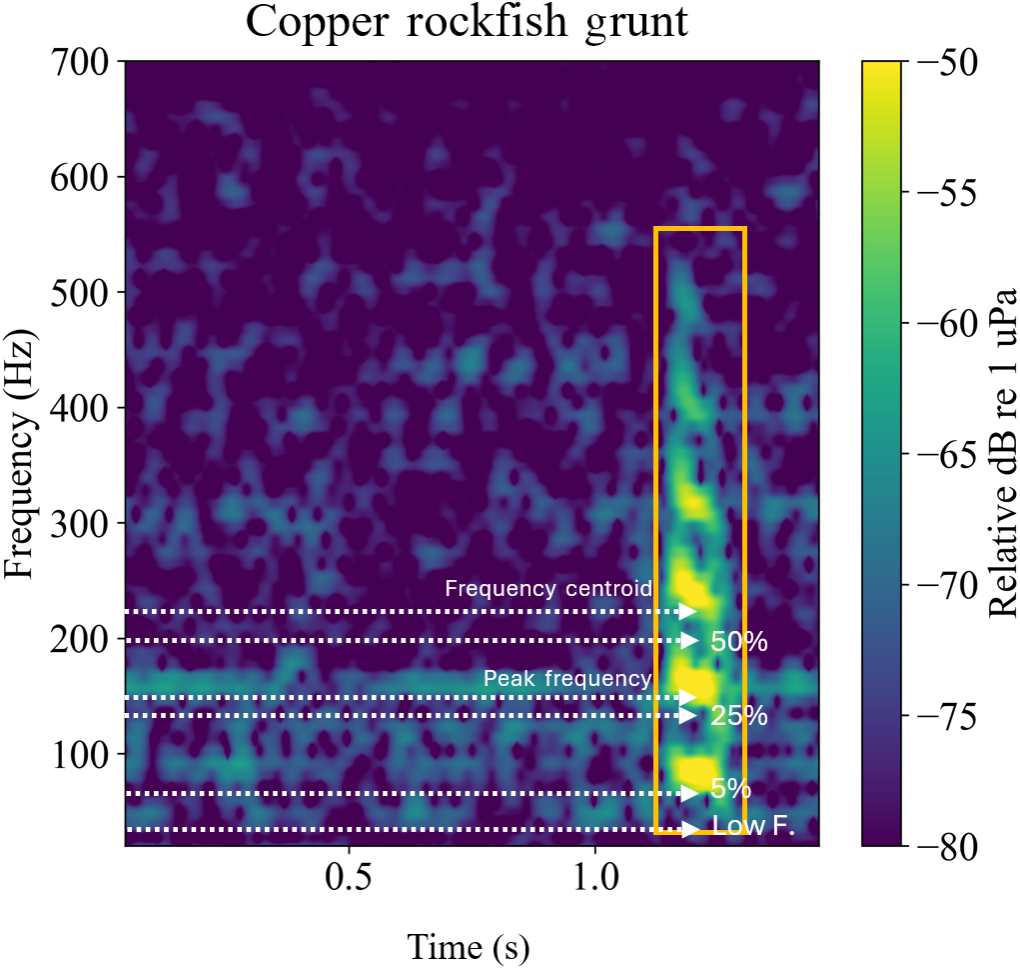
Visualization of the location of the top six sound features for differentiating fish grunts. Example spectrogram shows locations of frequency features on a single copper rockfish (*Sebastes caurinus*) grunt. Percentages represent frequency features (i.e. frequency 25%). Sound selection boxes are shown in yellow.

Correct classification of sounds as black and copper rockfish grunts was high with F1 scores >0.9 for both species (Table 5). Quillback rockfish call classification had lower precision and recall but still performed well (F1 score = 0.73). Frequency 25%, a measure of lower frequency energy skewness, was the most important sound feature for classifying grunts using the Gini impurity index (Figure 9 and Table S4).

**TABLE 5:** Random forest results from grunt test dataset.

Grunt class	Precision	Recall	F1 score	Prevalence	Detection rate	Detection prevalence	Balanced accuracy	*n*
Black rockfish	0.92	0.92	0.92	0.39	0.36	0.39	0.94	13
Copper rockfish	0.93	0.87	0.90	0.45	0.39	0.42	0.91	15
Quillback rockfish	0.67	0.80	0.73	0.15	0.12	0.18	0.86	5

*Note*: Full species scientific names: black rockfish (*Sebastes melanops*), copper rockfish (*Sebastes caurinus*), quillback rockfish (*Sebastes maliger*).

**FIGURE 10:**
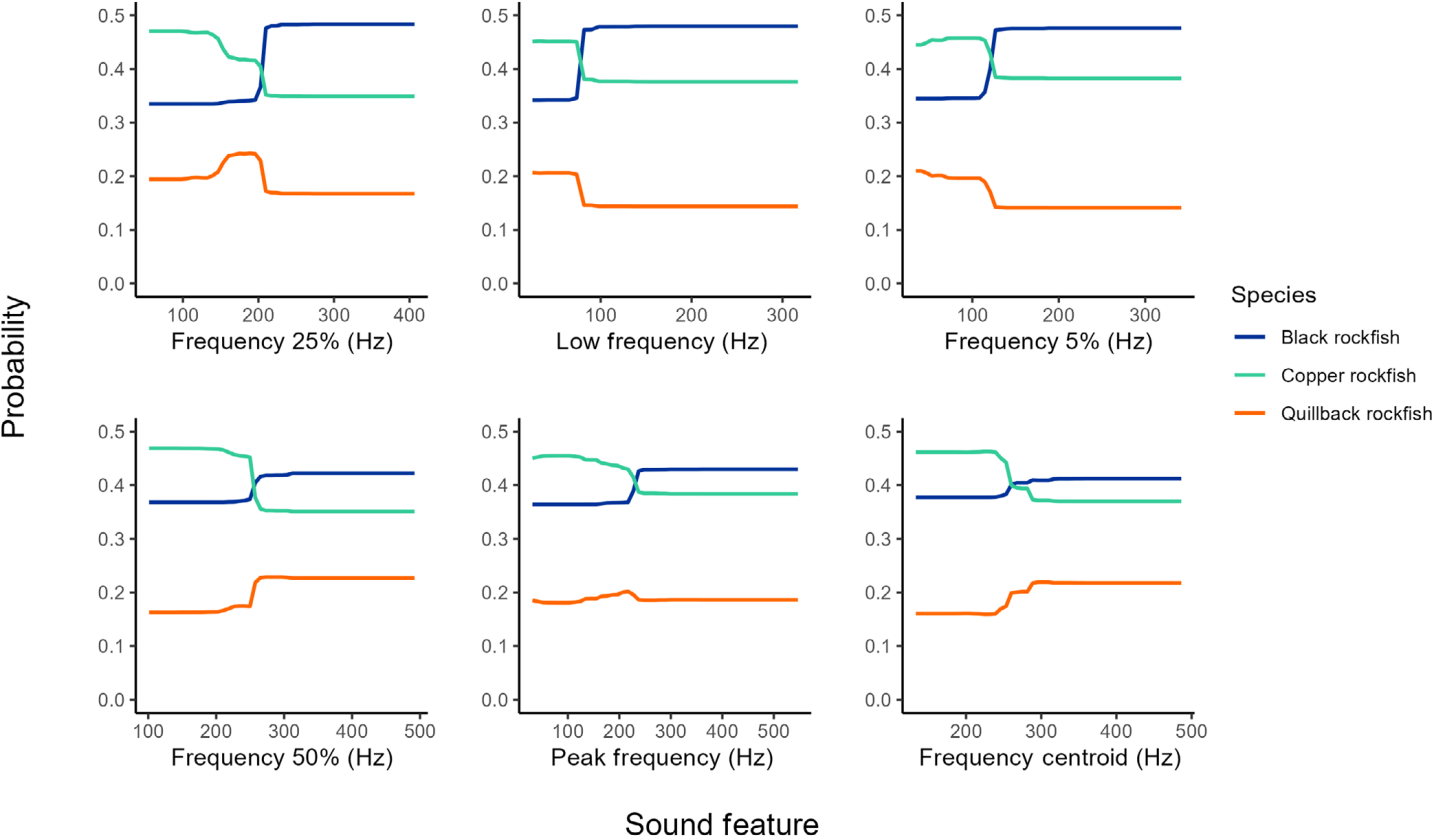
Partial dependence probability plots for the top six grunt sound features (Gini impurity Index). Full species scientific names: black rockfish (*Sebastes melanops*), copper rockfish (*Sebastes caurinus*), quillback rockfish (*Sebastes maliger*).

PDP plots showed that the probability of a grunt being from a black rockfish increased when frequency 25% was over ~200 Hz (Figure 10). Copper rockfish grunt probability decreased when frequency 25% was over ~150 Hz and quillback rockfish grunt probability spiked when frequency 25% was between ~150 and ~200 Hz. Black and copper rockfish grunt probability showed inverse trends across key frequencies. Copper and quillback rockfish grunt probability show inverse trends for frequency 50% and frequency centroid with probability increasing for quillback rockfish and decreasing for copper rockfish at ~250 Hz (Figure 10).

**FIGURE 11:**
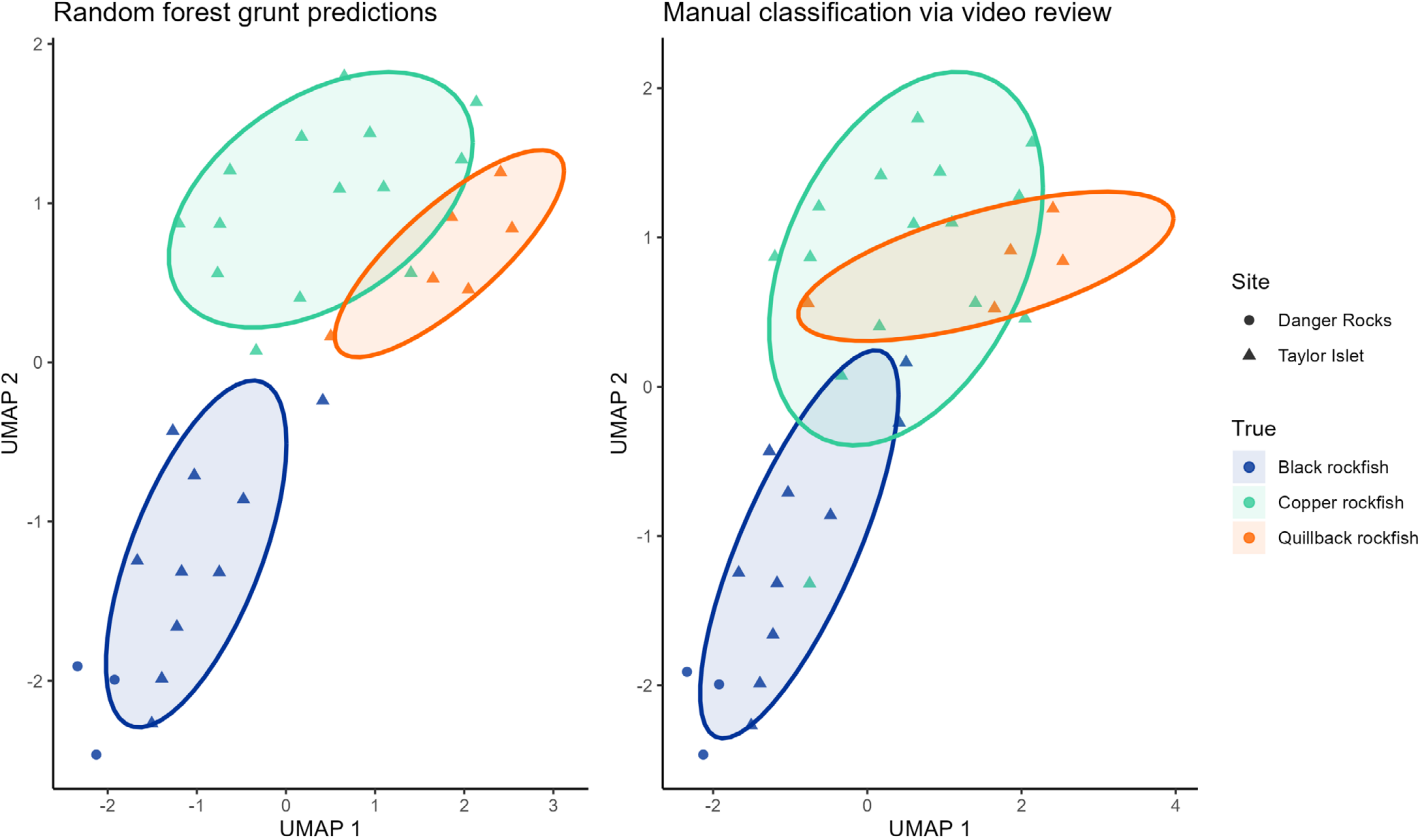
Uniform manifold approximation and projection visualization of grunt sound feature groupings coloured by species class with 70% confidence level ellipses. Full species scientific names: black rockfish (*Sebastes melanops*), copper rockfish (*Sebastes caurinus*), quillback rockfish (*Sebastes maliger*).

UMAP plots did not show site‐based trends in the random forest grunt classification, but black rockfish grunts from Danger Rocks were spaced further from grunts from Taylor Islet, suggesting site‐based, background noise differences are detectable in UMAP clustering (Figure 11). Black rockfish grunts show separation from other species' grunts, and copper and quillback rockfish show some overlap in both the random forest classification and UMAP clustering.

## 4 DISCUSSION

In this study we successfully identified unique fish species calls using passive acoustic localization data collected in the wild for a group of closely related rocky reef fishes. We used random forest models to define key sound features for parameterizing future species‐specific fish sound detectors (see Tables S3 and S4 for full species parameters). These methods could be applied in fish habitats elsewhere because the 47 sound features we used to train our models can classify sound differences across a broad range of temporal and frequency features. The most important sound features for differentiating fish sounds were frequency‐based features. Temporal features like call duration lacked differentiation power and ranked low in variable importance plots (Tables S1 and S2), especially for knocks that are short sounds of similar duration. Measures of low‐frequency tendencies like frequency 5% and 25% and measures of central frequency characteristics like frequency centroid and frequency 50% were important features for differentiating both knocks and grunts. These sound features, measured from the 95% energy spectral envelope (Mouy et al., 2024), are more robust to selection box variability than features like high and low frequency, which can be impacted by annotator variability, outliers and background noise (Cortopassi, 2006; Mellinger & Bradbury, 2007). Researchers looking to differentiate fish species calls should consider using these robust sound features in classification models and detectors rather than relying on basic sound measurements like high and low frequency. We recommend researchers conducting similar studies also use this random forest method as these models can effectively identify small differences in large, complex acoustic datasets that can be challenging to interpret manually using traditional sound measures (Rountree & Juanes, 2020; Širović et al., 2009).

We documented knocks and/or grunts for eight species of rocky reef fish. Two species—canary and vermillion rockfish—have never been documented as soniferous, and pile perch sounds have not been documented since 1966 (Meldrim & Walker, 1966). Black rockfish sounds have never been described or presented in spectrogram form (Fletcher, 1969). We provide summaries of species sound features in Tables S3–S5 and audio of species sounds are available on our data repository. We also demonstrate the importance of analysing field rather than aquarium recordings to determine if fish are soniferous and to characterize species calls. Aquarium‐based studies can struggle to determine sonifery and the range of sounds in fishes' repertoires. For example, an aquarium study by Nichols (2005) failed to elicit sounds from canary, black, and vermillion rockfish through prodding, but our study in fish habitat found that all three species are soniferous.

The random forest knock model performed well for differentiating black, canary and copper rockfish knocks but could not confidently classify knocks for lingcod, pile perch or quillback rockfish, which had the lowest sample sizes. Increasing knock sample size for these species would likely improve model performance. Vermillion rockfish knocks were not included in the random forest model due to low sample size, but an examination of their sound features (e.g. very small bandwidth, very low frequency; Figure 4) suggests that these calls could be confidently classified in a fish sound detector. Classifying closely related species knocks may be challenging as many knock sound features overlap. Future work on species‐specific fish sound detectors should incorporate information from the full repertoire of known fish sounds (e.g. grunts and knocks) to help differentiate species calls. Copper and quillback rockfish grunts were more easily differentiated than knocks so a fish sound detector that classifies both knocks and grunts would produce more accurate estimates of species presence.

We did not examine call repetition patterns in this paper because most PAM studies lack the localization and video data required to link repeated calls to individual fish. To increase the utility of this research for future PAM applications, our goal was to determine if individual fish species calls were distinct enough to develop a species‐specific fish sound detector without information on repeated calling patterns. Our work shows that many species calls like canary and black rockfish sounds can be differentiated with high confidence, suggesting a species‐specific fish sound detector is feasible for multiple species. However, the overlap of some species sounds suggests further research on call repetition could improve detector performance. Most bird sounds are identified using complex analyses of song structure rather than single, short chirps (Vidaña‐Vila et al., 2017; Zhao et al., 2017). It is likely that there are similar species‐specific nuances to fish vocalizations (Amorim, 2006). A study of piranhas in Peru by Rountree and Juanes (2020) found mean call repetition varied from two to 11 barks across different species, and bark interval showed species‐specific variation.

We did not include waveform measurements (Figures S3 and S4) in our analysis and instead focused on measurements that could be easily automated. The temporal envelope analysis we use captures some waveform‐related features such as duration, temporal concentration, asymmetry, kurtosis and roughness, but does not capture the finer measurements such as pulse frequency and pulse repetition rate. A manual analysis of fish sound waveforms by Mouy et al. (2023) found that lingcod, copper rockfish and quillback rockfish showed measurable differences in pulse frequency and pulse duration. However, there is currently no method to automate these waveform measurements and manual analysis of each call was beyond the scope of this project. Future work should examine detailed waveform measurements like pulse frequency and repetition as they would likely improve species classification accuracy.

It was not possible to determine if kelp greenling are soniferous during this study. Kelp greenling were frequently present in videos, and we identified 25 possible kelp greenling calls with low ID confidence. Kelp greenling were typically interacting with other soniferous fish during calling activities so it was impossible to confidently determine which fish was calling. Further studies on kelp greenling calling would be useful to determine if they are soniferous.

Copper and quillback rockfish showed similar sound feature characteristics and were sometimes misclassified in the random forest knock model. This similarity is unsurprising as these two species can hybridize (Schwenke et al., 2018). However, quillback rockfish knocks and grunts had higher peak frequencies than copper rockfish sounds. We are unsure if this is a species‐specific trait or an artefact of size differences across species. Higher frequency sounds often occur in smaller conspecifics (Kasumyan, 2008; Mann & Lobel, 1995; Myreberg et al., 1993; Rountree & Juanes, 2020), and larger copper rockfish are typically found at the same depth range as smaller quillback rockfish (Love et al., 2002). Future work will use our stereo‐camera length information to examine size impacts on call characteristics.

This study expands the utility of PAM for assessing species richness and presence/absence, which are cornerstones of conservation and fisheries monitoring. We outline a novel method for collecting wild fish sounds and identifying species‐specific sound features for use in fish sound detectors. Our study results can be used to detect the presence of specific fish species based on our documented sound parameters, which provide much greater precision than acoustic indices like ADI (Dimoff et al., 2021; Minello et al., 2021). Our work also contributes to the growing library of marine fish sounds required for PAM abundance estimation, but more localization studies are required to document the diversity of soniferous fish sounds. Future research should focus on localizing sounds for more species as well as collecting additional sound samples for underrepresented species. Further research into regional differences in species‐specific calls is also recommended to determine the transferability of sound characteristics.

## AUTHOR CONTRIBUTIONS

D.L.: Conceptualization, methodology, data acquisition, data curation, data analysis, visualization, writing‐original draft, writing‐review, editing. X.M.: Conceptualization, supervision, methodology, data acquisition, data analysis, writing‐review, editing. D.H.: Conceptualization, supervision, data acquisition, funding acquisition, writing‐review, editing. F.J.: Conceptualization, supervision, funding acquisition, writing‐review, editing.

## FUNDING INFORMATION

This research was supported by a Canadian Science Research Fund (CSRF) grant and a Natural Sciences and Engineering Research Council of Canada (NSERC) Canadian Graduate Scholarship.

## CONFLICT OF INTEREST STATEMENT

The authors declare no conflicts of interest.

## Supporting information


**Data S1.** Supporting Information.

## Data Availability

The data and code used in this study are available on Borealis https://doi.org/10.5683/SP3/MBT82V.
