# Supplementary material for: Knock knock, who's there? Identifying wild species‐specific fish sounds with passive acoustic localization and random forest models
Source: J Fish Biol. 2025 Dec 3;108(6):1646–60. doi: 10.1111/jfb.70294 (PMC13357343; doi:10.1111/jfb.70294)

**Supplementary Data - Knock knock, who’s there? Identifying wild species-specific fish sounds with passive acoustic localization and random forest models**

Table 1. Knock random forest classification results from training dataset. Error represents error rate for each class in the random forest calculated as - # misclassified calls per class/total calls per class.


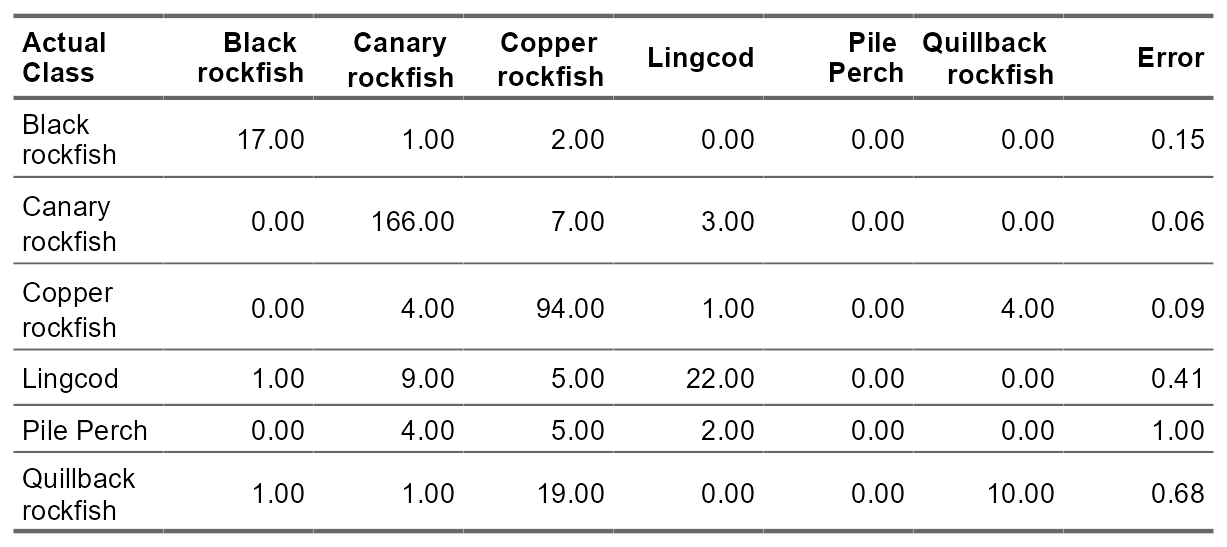


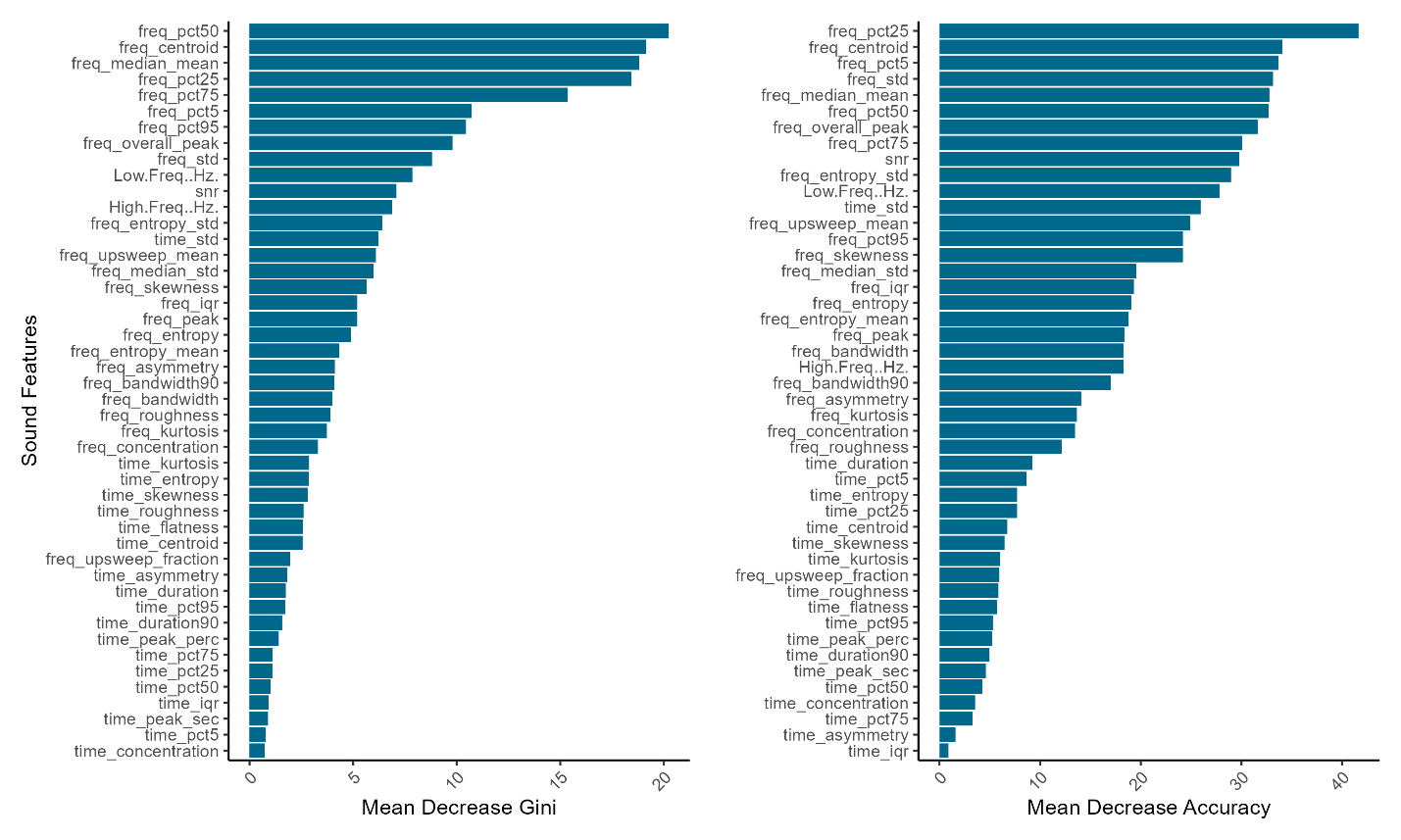


Figure 1. Variable importance plots for knock sound features.

Table 2. Grunt random forest classification results from training dataset. Class.error represents error rate for each class in the random forest calculated as - # misclassified calls per class/total calls per class.


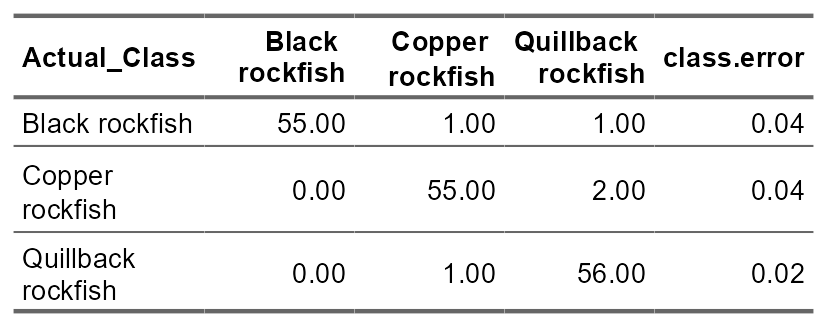


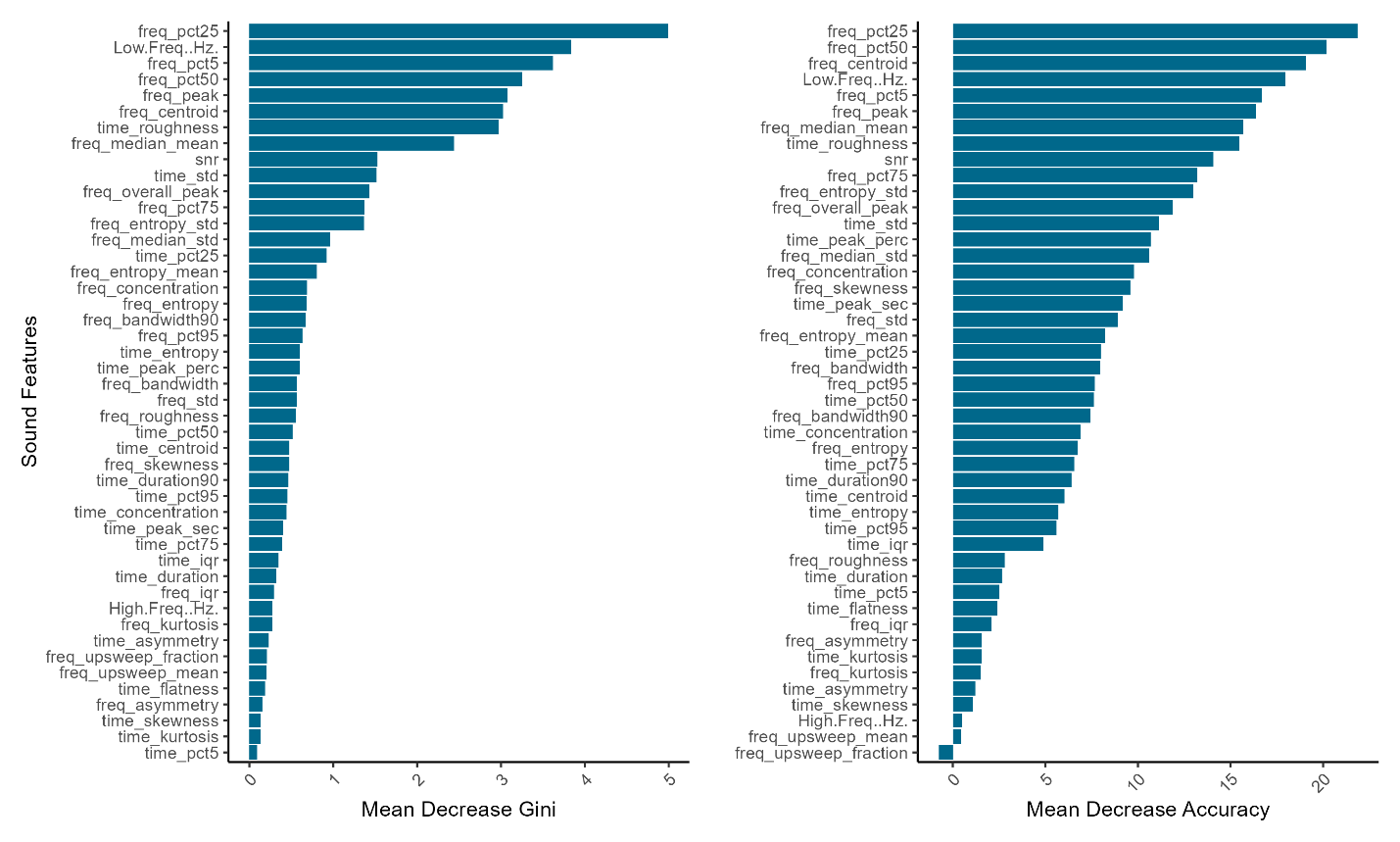


Figure 2. Variable importance plots for grunt sound features.

Table 3. Full summary of sound features for knocks used in random forest classification for each fish species. Numbers after +/- denote standard deviation.
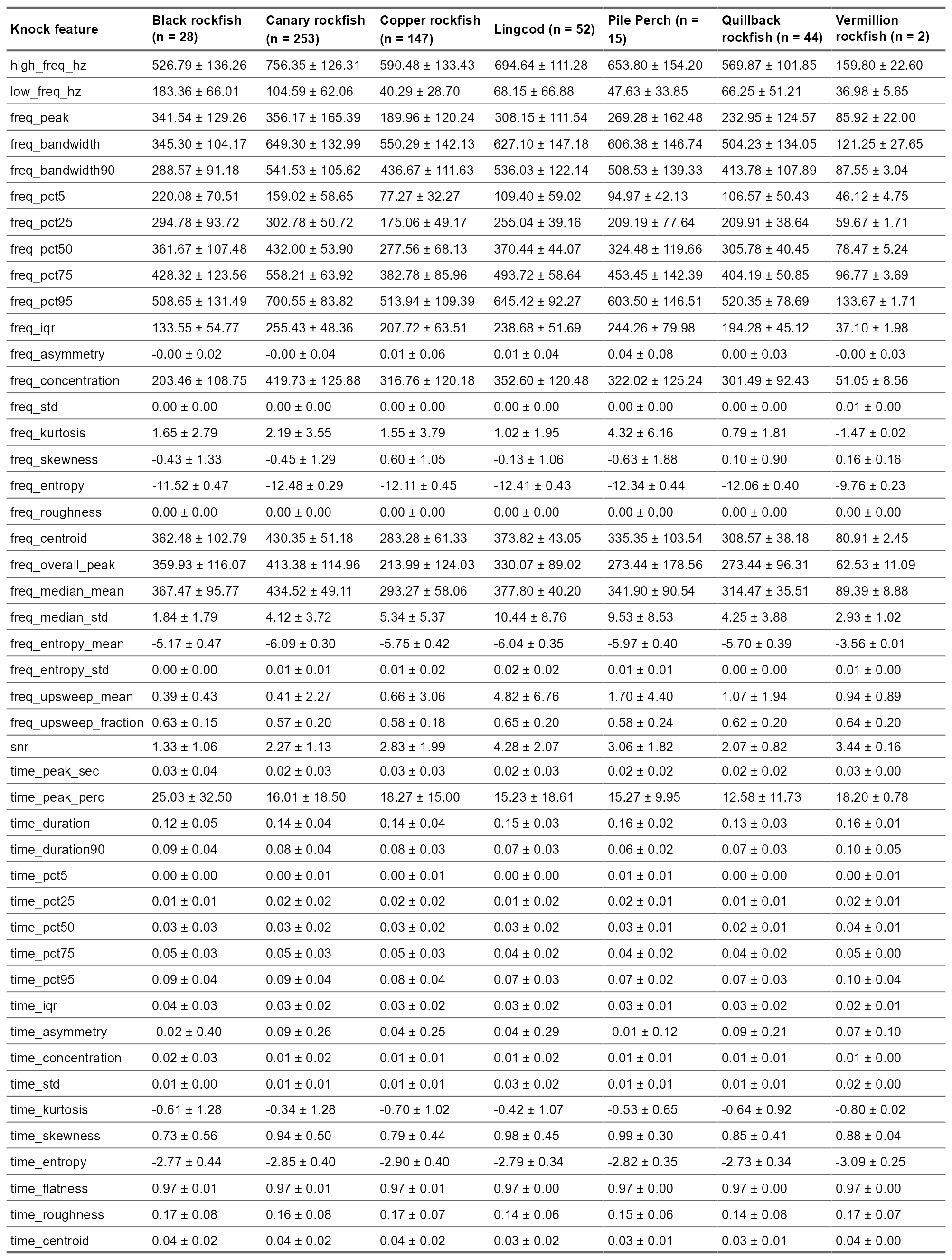


Table 4. Full summary of sound features for grunts used in random forest classification for each fish species. Numbers after +/- denote standard deviation.
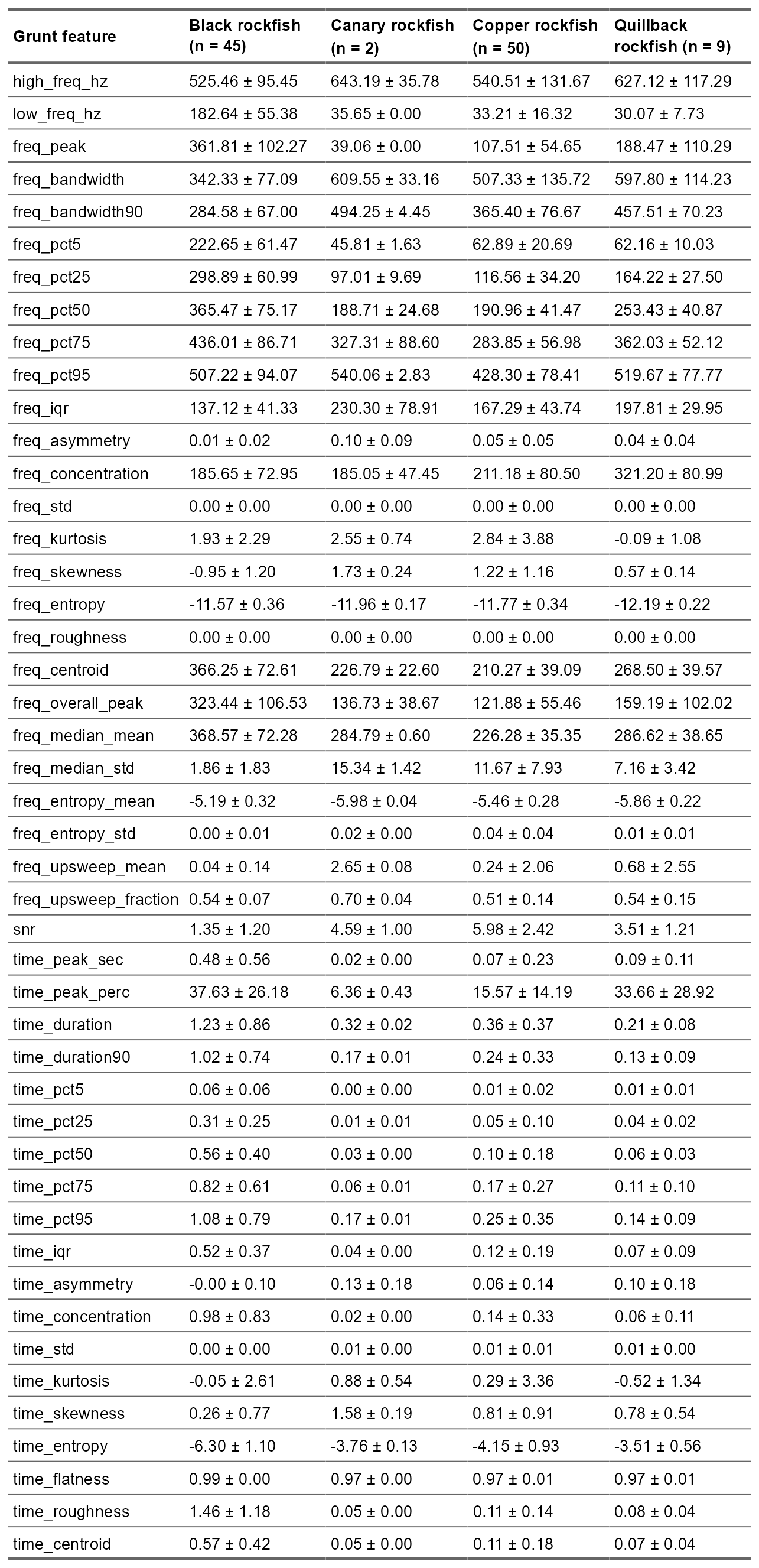


Table 5. Full summary of sound features for other sounds used for each fish species. Numbers after +/- denote standard deviation. Other sounds were not included in random forest analyses due to low sample numbers.


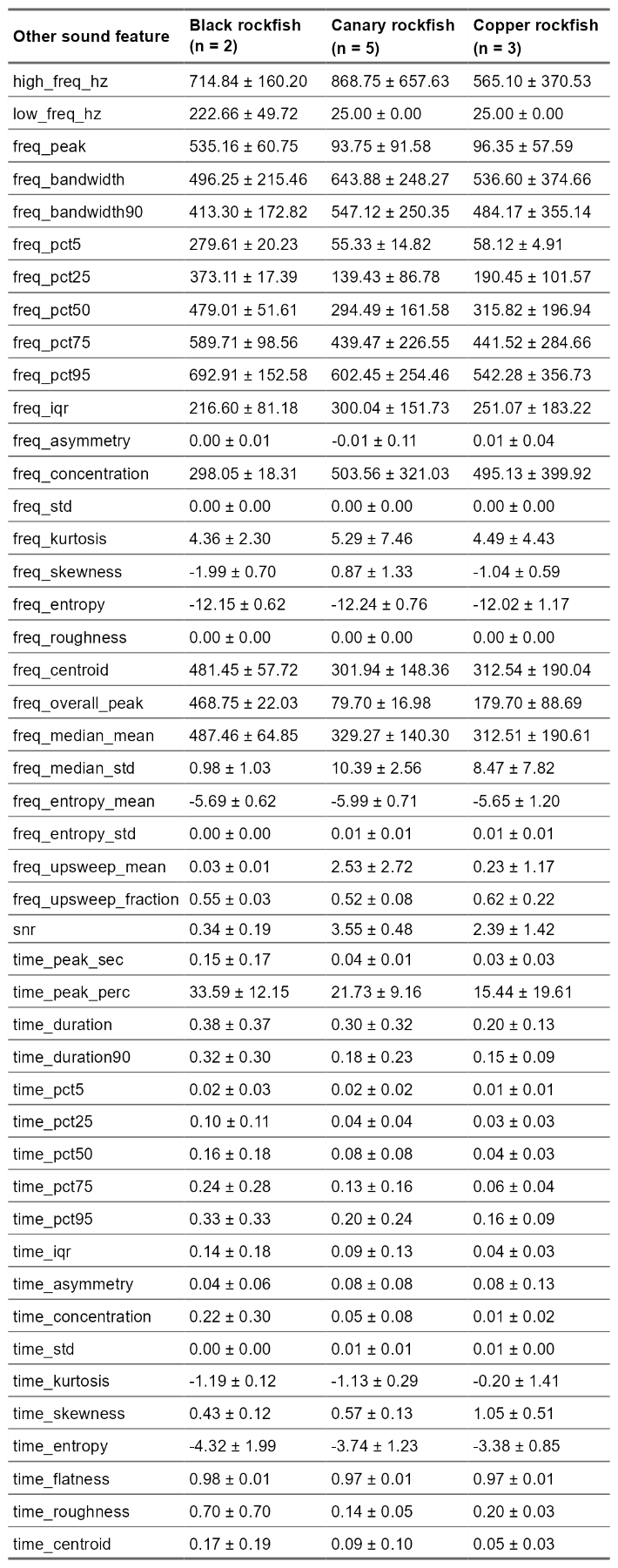


Table 6. Summary of other (non-grunt/knock) sounds (mean and standard deviation) for each species. Only sounds with an ID confidence of 1 were included.


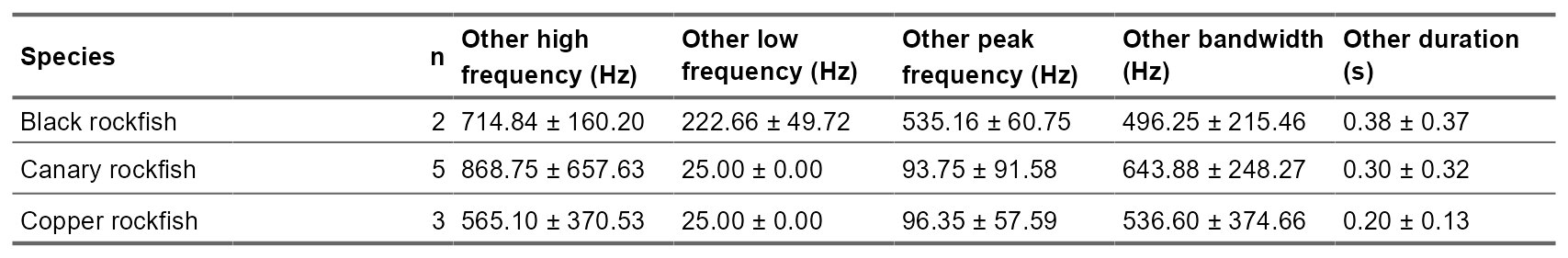


Figure 3. Knock waveforms for individual knocks for all identified fish species. Audio files were smoothed using down sampling (1 point per 10 samples).


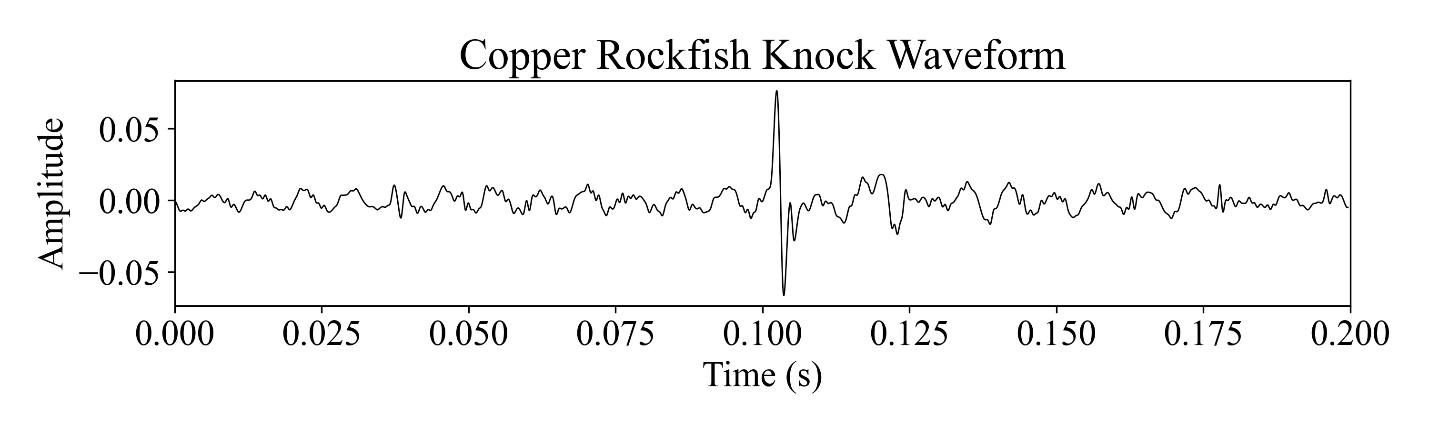

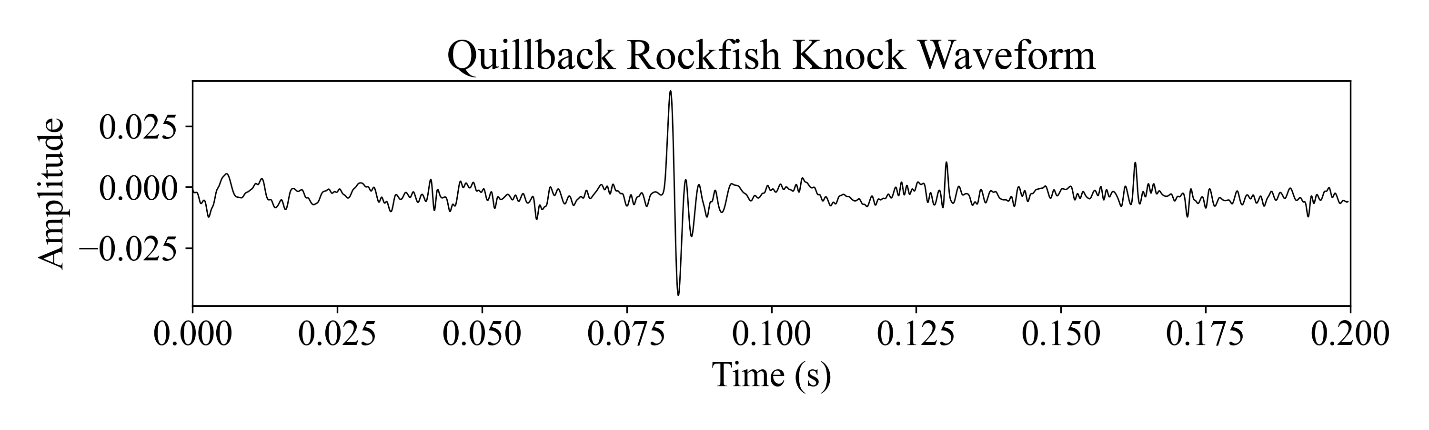

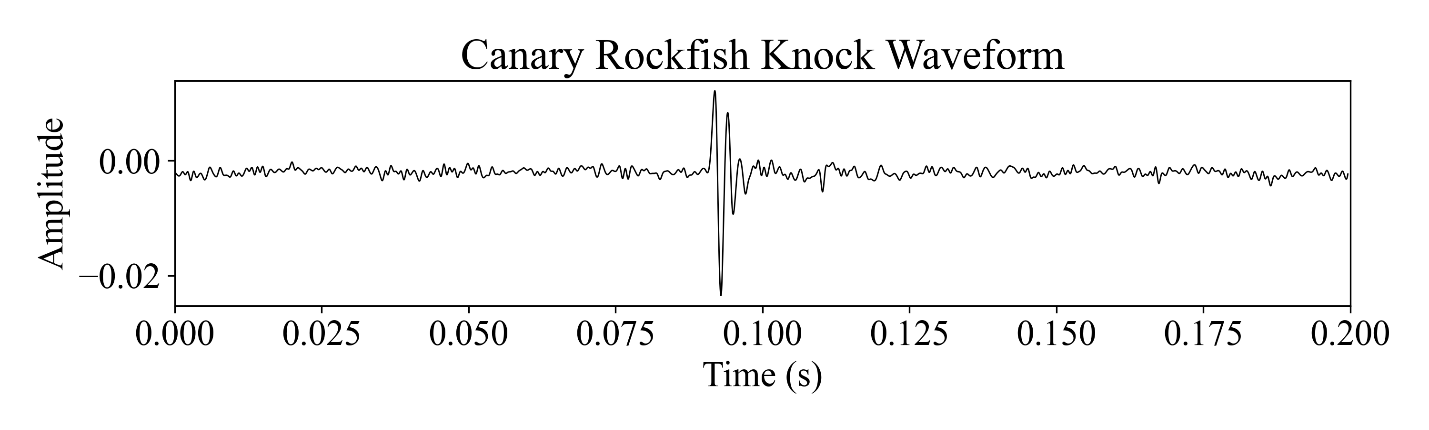


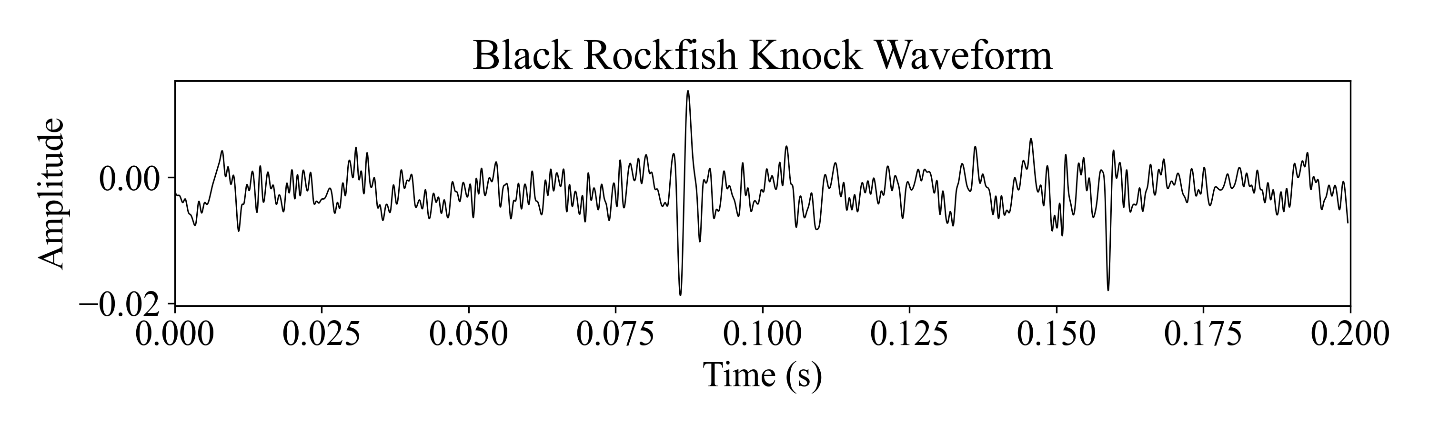

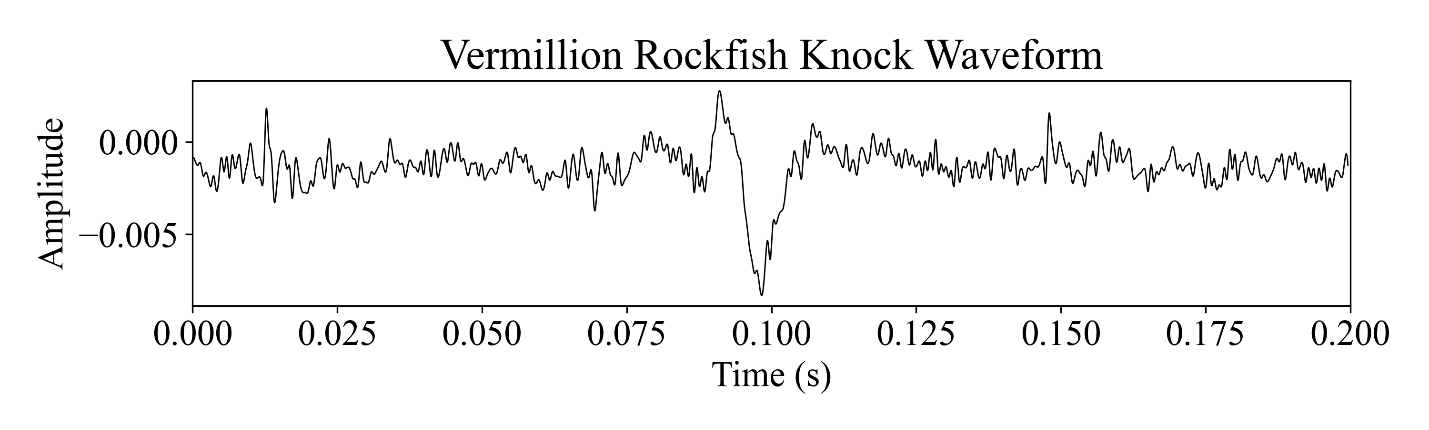

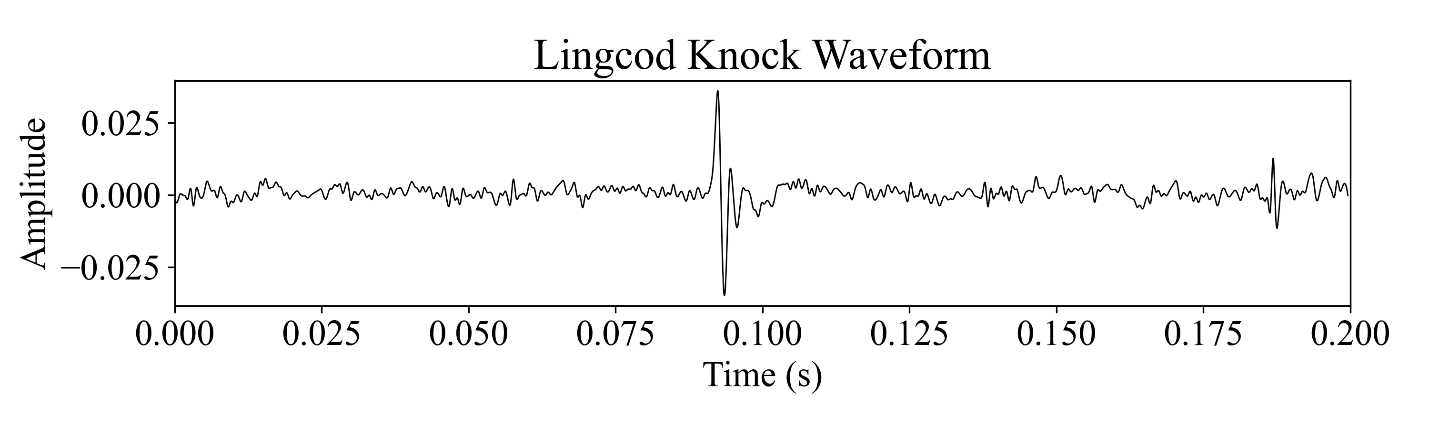

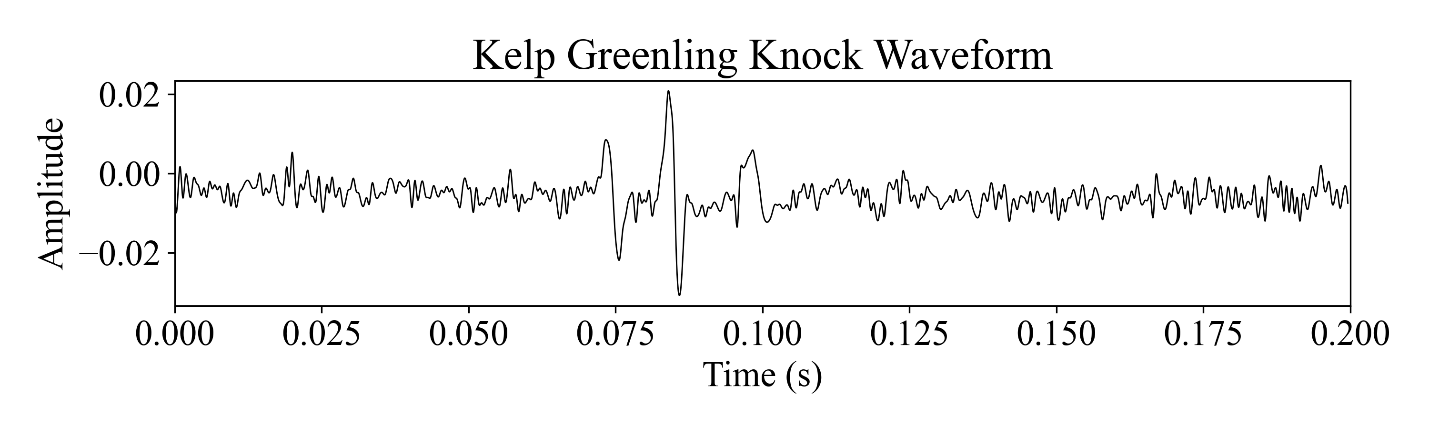

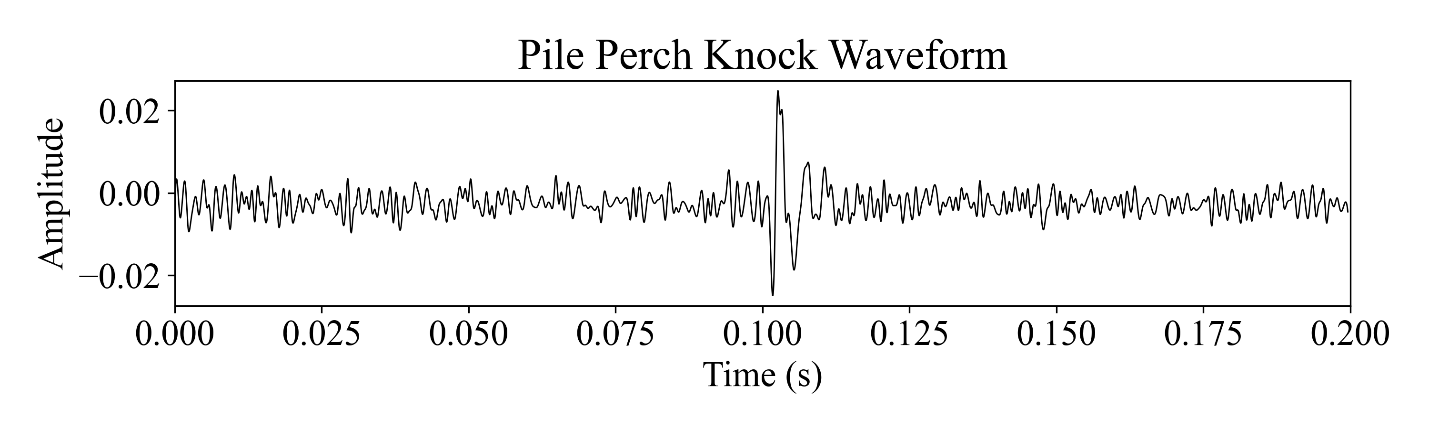


*Figure 4. Grunt waveforms for individual grunts for all identified fish species. Audio files were smoothed using down sampling (1 point per 10 samples).*


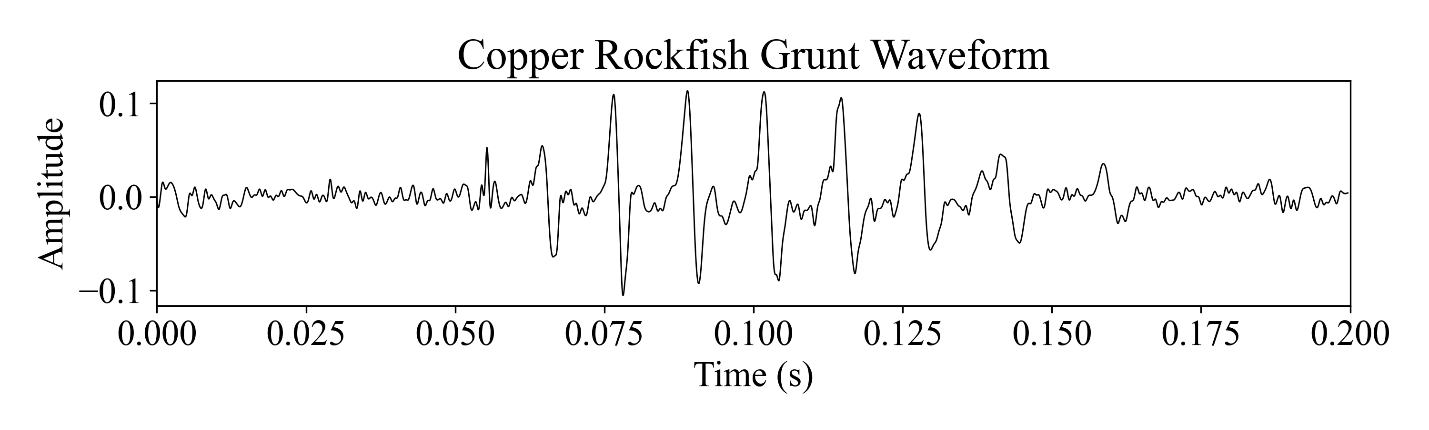

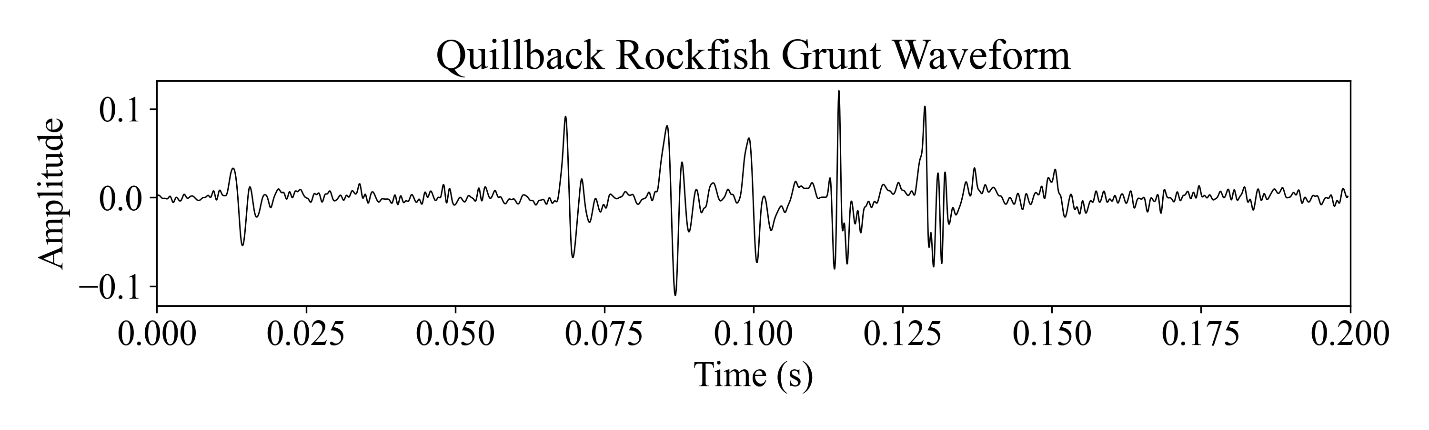

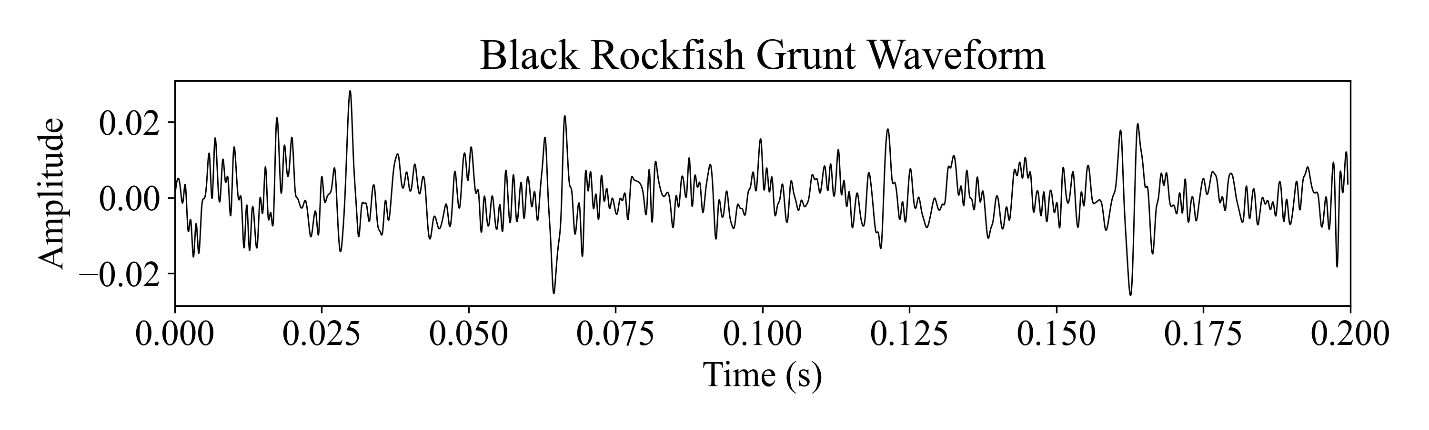

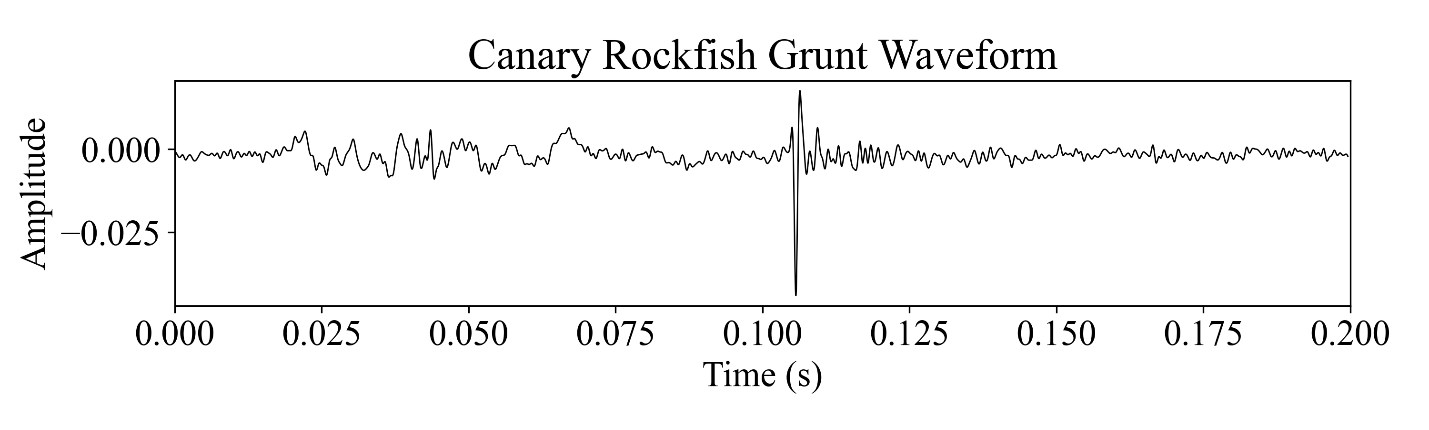

Supplement: Supplementary file 1 — Data S1. Supporting Information. [file JFB-108-1646-s001.docx]
